# Global Burden and Changing Trend of Hepatitis C Virus Infection in HIV-Positive and HIV-Negative MSM: A Systematic Review and Meta-Analysis

**DOI:** 10.3389/fmed.2021.774793

**Published:** 2021-12-13

**Authors:** Yang Zheng, Meike Ying, Yuqing Zhou, Yushi Lin, Jingjing Ren, Jie Wu

**Affiliations:** ^1^State Key Laboratory for Diagnosis and Treatment of Infectious Diseases, National Clinical Research Center for Infectious Diseases, Collaborative Innovation Center for Diagnosis and Treatment of Infectious Diseases, The First Affiliated Hospital, School of Medicine, Zhejiang University, Hangzhou, China; ^2^Department of General Practice, The First Affiliated Hospital, School of Medicine, Zhejiang University, Hangzhou, China; ^3^Department of Respiratory Medicine, Sir Run Run Shaw Hospital, School of Medicine, Zhejiang University, Hangzhou, China

**Keywords:** hepatitis C virus (HCV), human immunodeficiency virus (HIV), men who have sex with men (MSM), prevalence, incidence, changing trend

## Abstract

**Background:** The disease burden of hepatitis C virus (HCV) infection in HIV-positive and HIV-negative men who have sex with men (MSM) is changing. We aim to provide an updated comprehensive estimate of HCV prevalence and incidence among the HIV-positive and HIV-negative MSM population at the country, regional, and global levels and their changing trends over time.

**Methods:** PubMed, Embase, PsycINFO, CINAHL, and conference databases were searched and eligible records on the prevalence and incidence of HCV antibodies were selected and pooled *via* a random-effects model. Meta-regression was performed to demonstrate the association between the pooled rates and study year.

**Results:** A total of 230 articles reporting 245 records from 51 countries with 445,883 participants and 704,249 follow-up person-years were included. The pooled prevalence of HCV in MSM was 5.9% (95% CI: 5.1–6.8), with substantial differences between countries and regions. Low- and lower-middle-income countries (12.3 and 7.0%) manifested a larger disease burden than high- and upper-middle-income countries (5.8 and 3.8%). HCV prevalence in HIV-positive MSM was substantially higher than in HIV-negative MSM (8.1 vs. 2.8%, *p* < 0.001). The pooled incidence of HCV was 8.6 (95% CI: 7.2–10.0) per 1,000 person-years, with an increasing trend over time, according to meta-regression (*p* < 0.05).

**Conclusion:** Global HCV prevalence in MSM varies by region and HIV status. Behavior counseling and regular HCV monitoring are needed in HIV-positive subgroups and high-risk regions. Given the upward trend of HCV incidence and sexual risk behaviors, there is also a continued need to reinforce risk-reduction intervention.

**Systematic Review Registration:** PROSPERO, identifier CRD42020211028; https://www.crd.york.ac.uk/prospero/.

## Introduction

Hepatitis C virus (HCV) infection is a global public health threat. It was estimated that there were 71 million persons living with chronic HCV infection worldwide and 1.75 million new HCV infections in 2015 (1). Acute HCV infection persists for ~6 months and 55–85% of patients with acute hepatitis C transition to chronic hepatitis C, which may cause a series of end-stage liver diseases, such as chronic liver disease, cirrhosis, and hepatocellular carcinoma, in ~20–30 years in some patients (2). Unsafe healthcare practices (including unprotected exposure to blood or blood products) in the past and injection drug use nowadays account for most HCV infections (3).

In men who have sex with men (MSM), an excess risk of sexual HCV transmission has commonly been reported, especially for those who are concurrently infected with HIV. For instance, since the year 2000, several studies have reported an expanding epidemic of sexually transmitted acute HCV infection in HIV-positive MSM in North America, Europe, Australia, and Asia (4, 5). Several factors may facilitate sexual transmission of HCV in HIV-positive MSM. HCV viral loads are higher in semen in the presence of HIV and a sufficient quantity of HCV is exposed to the rectum in HIV-infected men, which may increase the infectivity of HCV carriers (6). In addition, behavioral factors such as increased high-risk sexual behavior and decreased precautionary measures are also believed to increase the HCV infection risk for HIV-positive MSM (7).

Despite a high HCV burden in HIV-positive MSM, HCV prevalence and incidence among HIV-negative MSM have been low and stable in previous studies, which is explained by the sexual behavior, sexual network different from those of HIV-positive MSM and other routes apart from sexual contact (8). Nevertheless, the most updated data revealed a high incidence of HCV in HIV-negative MSM (9). In addition, the identification of sexual network overlap among HIV-positive and HIV-negative MSM that placed HIV-negative MSM at high risk for HCV infection, as suggested that HIV-negative MSM is infected with HCV strains already circulating among HIV-positive MSM (10).

Considering the high HCV incidence and sexual network alternation in the recent years among the MSM population, the overall global HCV burden is expected to substantially change. However, there were few studies reported the updated comprehensive quantitative review of the global HCV burden in the MSM population. A recent meta-analysis focused on the HCV in both the HIV-positive and HIV-negative MSM population between 2000 and 2019, yet it only revealed the prevalence and incidence without further demonstration of their changing trends (11). Therefore, we conducted this updated systematic review and meta-analysis to provide a comprehensive estimate of HCV prevalence and incidence among the HIV-positive and HIV-negative MSM population at the country, regional, and global levels and their changing trends between 1990 and 2021.

## Methods

### Literature Search and Study Inclusion

This systematic review and meta-analysis were conducted adhering to the Preferred Reporting Items for Systematic Reviews and Meta-Analyses (PRISMA) 2009 guidelines (12). We prospectively submitted the systematic review protocol for registration on the PROSPERO (CRD42020211028). We comprehensively searched the traditional databases of PubMed, Embase, PsycINFO, and CINAHL using a combination of medical subject headings and free text including terms related to HCV, MSM, and prevalence. We also searched HIV/AIDS and hepatology-related conference databases (e.g., International AIDS Society Conference, Conference on Retroviruses and Opportunistic Infections, American Association for the Study of Liver Diseases, European Association for the Study of the Liver) (Supplementary Appendix 1). All the related published papers from January 1, 1990 to July 31, 2021 were identified and subsequently stored using EndNote X9.

Sources were eligible for inclusion; if the study population was exclusively MSM or if MSM could be separated from other population; if the researchers conducted serological testing for anti-HCV and/or HCV RNA; and if the study presented an estimate of anti-HCV prevalence or HCV incidence (defined as antibody seroconversion or positive RNA). Incidence sources were restricted to those in which seroconversion was known to have occurred. Sources were excluded; if the study population was a transgender population; if HCV infection status was self-reported or no information with respect to HCV status was determined; if sources had a sample size of less than 30 or had no information with respect to sample size; if it was HCV reinfection; and if the study was a case report, case series, or review. There were no restrictions on language of publication or the age of the sampled population. Multiple publications with the same data source were excluded with detained the most recent publication.

### Data Extraction

Two authors (Zheng Y and Ying M) screened all the sources for inclusion, with a third senior reviewer (Wu J) consulted when disagreement occurred. A review of potentially eligible reports was a multistage process, as depicted in the PRISMA flow diagram. We extracted the following information for all the eligible studies: the first author; publication year; country and city of the study; years of study start and end; study design; sample size or follow-up person year (FUPY) of HCV; number of HCV-positive and HCV-negative people; HCV testing methods; and number of concurrent HIV-positive and HIV-negative people.

### Outcomes

To calculate the prevalence of HCV in MSM, we extracted the number of HCV positive and total number of HCV tested. Based on the available literature, we also performed a subgroup analysis to estimate the prevalence of HCV in HIV-positive and HIV-negative MSM. HCV incidence was calculated from subgroups of studies that followed MSM without HCV at baseline for the development of HCV, in which the number of HCV positive and total FUPY were extracted. Country-level pooled rates were further aggregated into the region-level and subregion-level rates of prevalence and incidence according to the United Nations Department of Economic and Social Affairs classification. We also examined the region-level rates of prevalence and incidence when countries were grouped by their income according to the World Bank classification (13).

### Quality Assessment

Included studies were assigned quality scores using the Newcastle–Ottawa Scale (NOS) for cohorts and case–control studies and a modified NOS for cross-sectional studies (14, 15). The NOS assesses three domains of methodology of study including study participant selection (0–4 points), confounder adjustment (0–2 points), and outcome indicator determination (0–3 points). Studies with the NOS score of 7–9 points were defined as high quality, while those with less than 7 points were deemed of low or medium quality (15).

### Statistical Analysis

We conducted a meta-analysis to calculate the pooled prevalence and incidence of HCV infection in MSM and their 95% CI using a random-effects model given the high heterogeneity expected. The Freeman–Tukey double arcsine transformation of the original positive rate was conducted to stabilize the variance to reduce the effect of extreme values on the pooled positive rate estimate (16). The results were presented as “percent” in the prevalence study and “per 1,000 person-years” in the incidence study with 95% CI and heterogeneity was assessed using *I*^2^. A subgroup analysis was performed to estimate the prevalence of HCV in subgroups by HIV status (positive or negative). We used meta-regression to investigate the changing trend of prevalence and incidence over time. The results were presented with bubble plots with a regression equation. Publication bias was assessed by Egger's test and funnel plot. Sensitivity analyses were conducted to assess the influence of each individual study on the final pooled estimates. Data were analyzed using the R software (version 4.0.0). *p* < 0.05 was deemed as statistically significant.

## Results

### Selection, Description, and Quality of Included Studies

**Figure 1:**
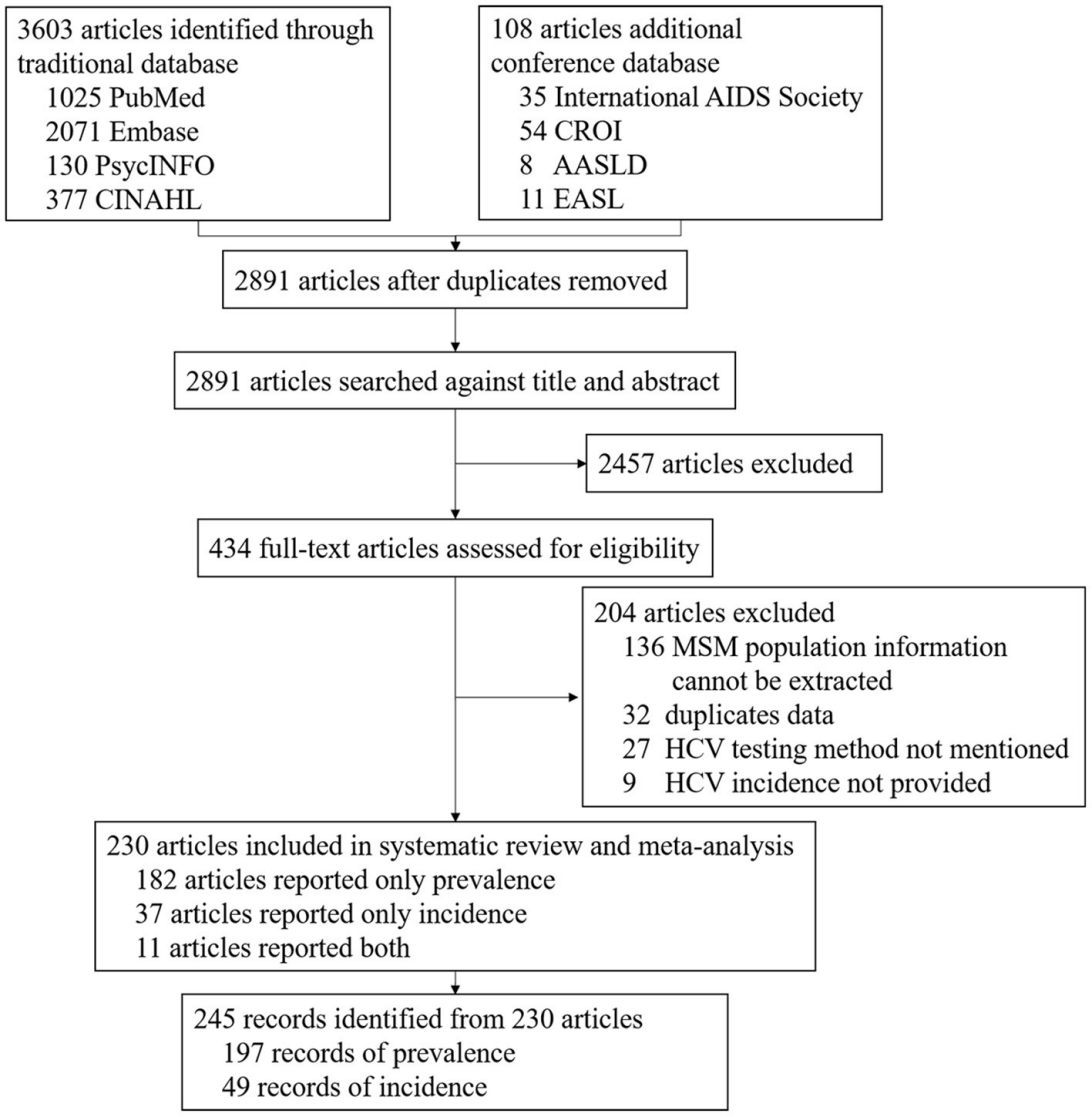
Flowchart of studies selection process.

A total of 3,711 articles were screened from the database, of which 820 duplicates were removed and 2,891 unique articles were remained. We excluded 2,457 articles that clearly did not meet the selection criteria by title or abstract screening. A total of 434 articles were assessed for full text. Among them, 204 articles were further excluded because MSM population information could not be extracted (*n* = 136), duplicated data (*n* = 32), HCV testing method not mentioned (*n* = 27), and HCV incidence not provided (*n* = 9). Finally, a total of 230 articles reporting 245 records from 51 countries with 445,883 participants and 704,249 FUPY were included after full-text review (Figure 1). Published year ranges from 1990 to 2021. Sample size ranged from 26 to 129,136 in prevalence studies and 282 to 315,392 FUPY in incidence studies. All the articles used HCV antibody test. Cross-sectional (*n* = 155) and cohort (*n* = 47) studies were the major study designs. A total of 94 records reported HCV infection in 54,530 HIV-positive MSM, while 49 records reported HCV infection in 45,937 HIV-negative MSM. Other characteristics of the included studies are shown in Supplementary Appendix 2.

### Hepatitis C Virus Prevalence and Changing Trend Over Time

**Figure 2:**
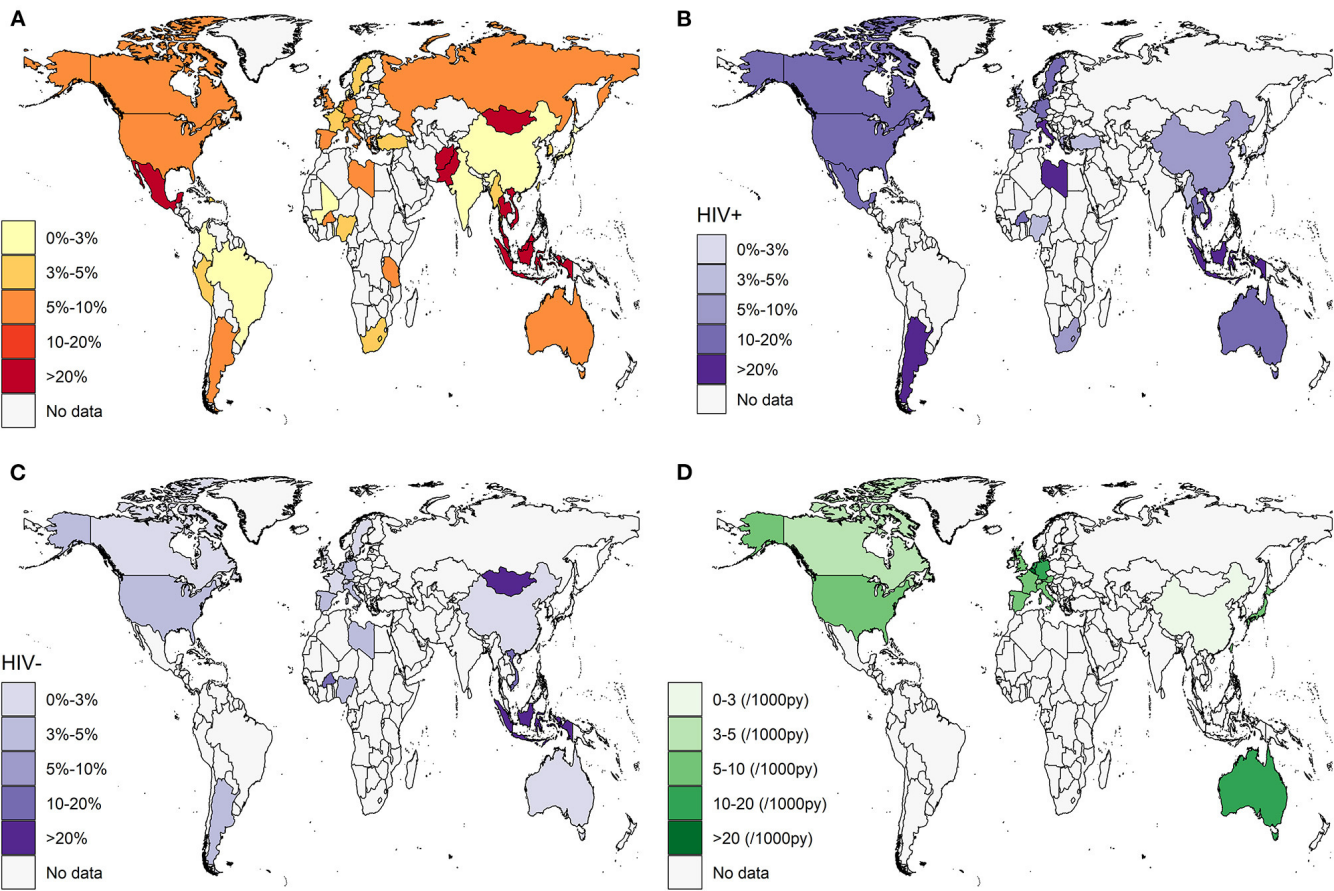
Estimated HCV prevalence and incidence in MSM by country. **(A)** HCV prevalence in overall MSM population; **(B)** HCV prevalence in HIV-positive MSM subpopulation; **(C)** HCV prevalence in HIV-negative MSM subpopulation; **(D)** HCV incidence in overall MSM population. White area indicated no data available. HCV, hepatitis C virus; MSM, men who have sex with men.

Across 196 records screening for HCV infection (anti-HCV positive) in 445,883 participants, the pooled prevalence of HCV in MSM was 5.9% (95% CI: 5.1–6.8) globally, with estimates ranging from 0.6 to 63.5% (*I*^2^ = 99%, Egger's test for bias, *p* < 0.05). The results of the publication bias and influential analysis are shown in Supplementary Appendices 3, 4. The omission of an individual study resulted in pooled estimates ranging from 5.7 to 6.0%. Table 1 and Figure 2A present the prevalence and number of reported participants by country. The countries with the highest HCV burden were Mexico (63.5%, 95% CI: 0.0–100.0), Malaysia (59.2%, 95% CI: 51.9–66.3), Thailand (41.4%, 95% CI: 4.6–85.3), Mongolia (36.0%, 95% CI: 23.2–49.9), and Indonesia (32.5%, 95% CI: 22.4–43.4). Countries with the lowest HCV prevalence were Togo (0.6%, 95% CI: 0.0–2.5), Mali (0.6%, 95% CI: 0.0–1.9), Lebanon (0.7%, 95% CI: 0.3–1.3), Brazil (0.8%, 95% CI: 0.3–1.5), and China (1.2%, 95% CI: 1.0–1.5). Regions with the highest reported HCV burden were Central America (63.5%, 95% CI: 0.0–100.0), Southeast Asia (29.0%, 95% CI: 14.4–46.1), and Southern Asia (15.1%, 95% CI: 0.3–44.5). Regions with the lowest reported HCV burden were Western Asia (1.3%, 95% CI: 0.0–4.6), Eastern Asia (1.9%, 95% CI: 1.6–2.3), and Western Africa (2.2%, 95% CI: 0.5–5.0). We found a substantial difference between high-/upper-middle-income countries (5.8 and 3.8%) and low-/lower-middle-income countries (12.3 and 7.0%), as shown in Table 2. Forest plots are given in Supplementary Appendix 5.

**Table 1:** Estimates of anti-HCV prevalence and incidence by geographic region.

**Geographic region**	**Prevalence**			**Incidence**		
	**Record**	**Participant**	**Pooled prevalence estimates, % (95% CI)**	**Record**	**Follow up person year**	**Pooled incidence estimates, /1,000 py (95% CI)**
**Asia**	**46**	**307,980**	**4.6 (3.8–5.5)**	**7**	**34,650**	**6.4 (2.0–13.0)**
**Eastern Asia**	**33**	**298,609**	**1.9 (1.6–2.3)**	**6**	**30,638**	**5.5 (1.0–13.3)**
China	20	284,596	1.2 (1.0–1.5)	1	507	2.0 (0.0–8.5)
Hong Kong *SAR*				1	6,295	2.2 (1.2–3.6)
Japan	1	1,068	2.1 (1.3–3.0)	1	2,246	9.4 (5.7–13.8)
Mongolia	1	50	36.0 (23.2–49.9)			
South Korea	1	320	4.1 (2.1–6.5)	1	1,550	2.6 (0.6–5.9)
Taiwan *Province*	10	12,575	3.2 (2.1–4.4)	2	20,040	9.7 (0.5–30.0)
**South-Eastern Asia**	**8**	**2,406**	**29.0 (14.4–46.1)**	**1**	**4,012**	**12.5 (9.2–16.1)**
Indonesia	1	77	32.5 (22.4–43.4)			
Malaysia	1	179	59.2 (51.9–66.3)			
Myanmar	1	177	3.4 (1.1–6.7)			
Singapore				1	4,012	12.5 (9.2–16.1)
Thailand	2	108	41.4 (4.6–85.3)			
Vietnam	3	1,865	22.6 (4.9–47.8)			
**Southern Asia**	**3**	**5,514**	**15.1 (0.3–44.5)**			
Afghanistan	1	124	30.6 (22.8–39.1)			
India	1	4,994	1.3 (1.0–1.6)			
Pakistan	1	396	23.5 (19.4–27.8)			
**Western Asia**	**2**	**1,451**	**1.3 (0.0–4.6)**			
Lebanon	1	1,351	0.7 (0.3–1.3)			
Turkey	1	100	3.0 (0.4–7.5)			
**Europe**	**84**	**86,525**	**5.3 (3.8–6.9)**	26^*^	**269,160**	**8.9 (7.0–11.1)**
**Eastern Europe**	**2**	**441**	**4.5 (0.9–10.1)**			
Moldova	1	391	3.3 (1.7–5.4)			
Russia	1	50	8.0 (1.8–17.4)			
**Northern Europe**	**26**	**25,999**	**5.1 (1.6–10.1)**	**4**	**89,721**	**7.6 (6.1–9.3)**
Denmark	2	397	1.5 (0.5–3.0)	1	3,484	4.0 (2.2–6.4)
Estonia	1	43	4.7 (0.1–13.5)			
Sweden	3	1,326	4.9 (0.0–16.2)			
United Kingdom	20	24,233	5.6 (1.6–11.7)	3	86,237	8.5 (7.9–9.1)
**Southern Europe**	**21**	**8,934**	**6.4 (4.6–8.6)**	**5**	**14,001**	**7.5 (4.0–11.9)**
Croatia	2	565	2.6 (1.4–4.2)			
Greece	1	124	8.1 (3.8–13.6)			
Italy	7	1,826	8.1 (4.8–12.2)	2	4,700	7.2 (0.7–19.5)
Spain	11	6,419	6.3 (3.7–9.4)	3	9,301	8.4 (3.2–16.0)
**Western Europe**	**35**	**51,151**	**4.7 (3.6–5.9)**	**16**	**146,510**	**10.2 (7.1–13.9)**
Austria	1	823	3.0 (2.0–4.3)	1	4,892	7.4 (5.1–10.0)
Belgium	3	641	3.9 (0.9–8.8)	1	318	28.3 (12.5–49.8)
France	8	15,783	4.2 (2.1–7.0)	4	59,728	7.4 (4.8–10.5)
Germany	5	8,731	9.7 (3.3–19.0)	1	6,054	16.4 (13.3–19.7)
Netherlands	9	8,311	4.9 (2.5–8.0)	8	51,811	11.5 (5.1–20.5)
Switzerland	9	16,862	3.2 (2.0–4.6)	1	23,707	4.3 (3.5–5.1)
**Africa**	**13**	**5,557**	**4.3 (2.3–6.9)**			
**Eastern Africa**	**3**	**1,427**	**9.6 (3.7–17.8)**			
Tanzania	3	1,427	9.6 (3.7–17.8)			
**Northern Africa**	**1**	**224**	**8.5 (5.2–12.5)**			
Libya	1	224	8.5 (5.2–12.5)			
**Southern Africa**	**3**	**1,572**	**3.4 (2.1–5.1)**			
South Africa	3	1,572	3.4 (2.1–5.1)			
**Western Africa**	**6**	**2,334**	**2.2 (0.5–5.0)**			
Burkina Faso	2	512	5.0 (0.0–18.6)			
Cote d'Ivoire	1	206	1.0 (0.0–2.9)			
Mali	1	320	0.6 (0.0–1.9)			
Nigeria	1	1,125	3.1 (2.2–4.2)			
Togo	1	171	0.6 (0.0–2.5)			
**Northern America**	**33**	**33,067**	**7.8 (5.9–9.9)**	**10**	**371,436**	**7.4 (5.3–9.9)**
Canada	9	5,618	9.1 (4.5–15.0)	3	11,269	4.5 (3.3–5.9)
United States	24	27,449	7.3 (5.3–9.7)	7	360,167	8.3 (5.6–11.5)
**Latin**						
**America/Caribbean**	**15**	**6,890**	**8.7 (4.1–14.8)**			
**Caribbean**	**2**	**1,429**	**2.7 (1.9–3.7)**			
Dominican Republic	1	1,388	3.2 (2.3–4.2)			
Puerto Rico	1	41	4.9 (0.1–14.1)			
**Central America**	**2**	**177**	**63.5 (0.0–100.0)**			
Mexico	2	177	63.5 (0.0–100.0)			
**South America**	**11**	**5,284**	**4.1 (2.3–6.3)**			
Argentina	6	2,668	6.5 (3.2–10.9)			
Brazil	3	1,117	0.8 (0.3–1.5)			
Colombia	1	1,100	2.1 (1.3–3.0)			
Peru	1	399	3.8 (2.1–5.9)			
**Oceania**	**5**	**5,864**	**7.6 (3.6–12.8)**	**6**	**29,033**	**11.8 (5.3–21.0)**
Australia	5	5,864	7.6 (3.6–12.8)	6	29,033	11.8 (5.3–21.0)
**Global**	**196**	**445,883**	**5.9 (5.1–6.8)**	**49**	**704,249**	**8.6 (7.2–10.0)**

* *There is a multi-center incidence study conduct in the whole Europe*.

*HCV, hepatitis C virus; CI, confidence interval*.

*Bold values represent results at the global or regional level, and non-bold values represent results at national level*.

**Table 2:** Estimated of anti-HCV prevalence and incidence by income level.

**World Bank region**	**High**	**Upper-mid**	**Lower-mid**	**Low**
	**income**	**income**	**income**	**income**
**Overall prevalence**				
Record	139	38	11	8
Participant	141,687	292,361	9,281	2,554
Pooled rate, % (95% CI)	5.8 (4.8–6.9)	3.8 (3.1–4.6)	12.3 (5.1–22.0)	7.0 (2.5–13.3)
**Subgroup prevalence in HIV+ MSM**				
Record	76	13	4	1
Participant	50,681	3,372	467	10
Pooled rate, % (95% CI)	7.7 (6.5–8.9)	8.2 (4.3–13.1)	37.6 (0.0–92.5)	10.0 (0.0–38.1)
**Subgroup prevalence in HIV– MSM**				
Record	39	5	4	1
Participant	25,904	17,276	2,438	319
Pooled rate, % (95% CI)	2.0 (1.2–3.0)	1.1 (0.4–2.1)	19.6 (6.1–38.1)	11.0 (7.8–14.7)
**Overall incidence**				
Record	47	1	/	/
Person year	684,814	507	/	/
Pooled rate, /1,000 py (95% CI)	8.7 (7.3–10.2)	2.0 (0.0–8.5)	/	/

*Country's income level is categorized into high income, upper-middle income, lower-middle income, and low income in accordance with the World Bank classification*.

*HCV, hepatitis C virus; CI, confidence interval; HIV, human immunodeficiency virus; MSM, men who have sex with men*.

**Figure 3:**
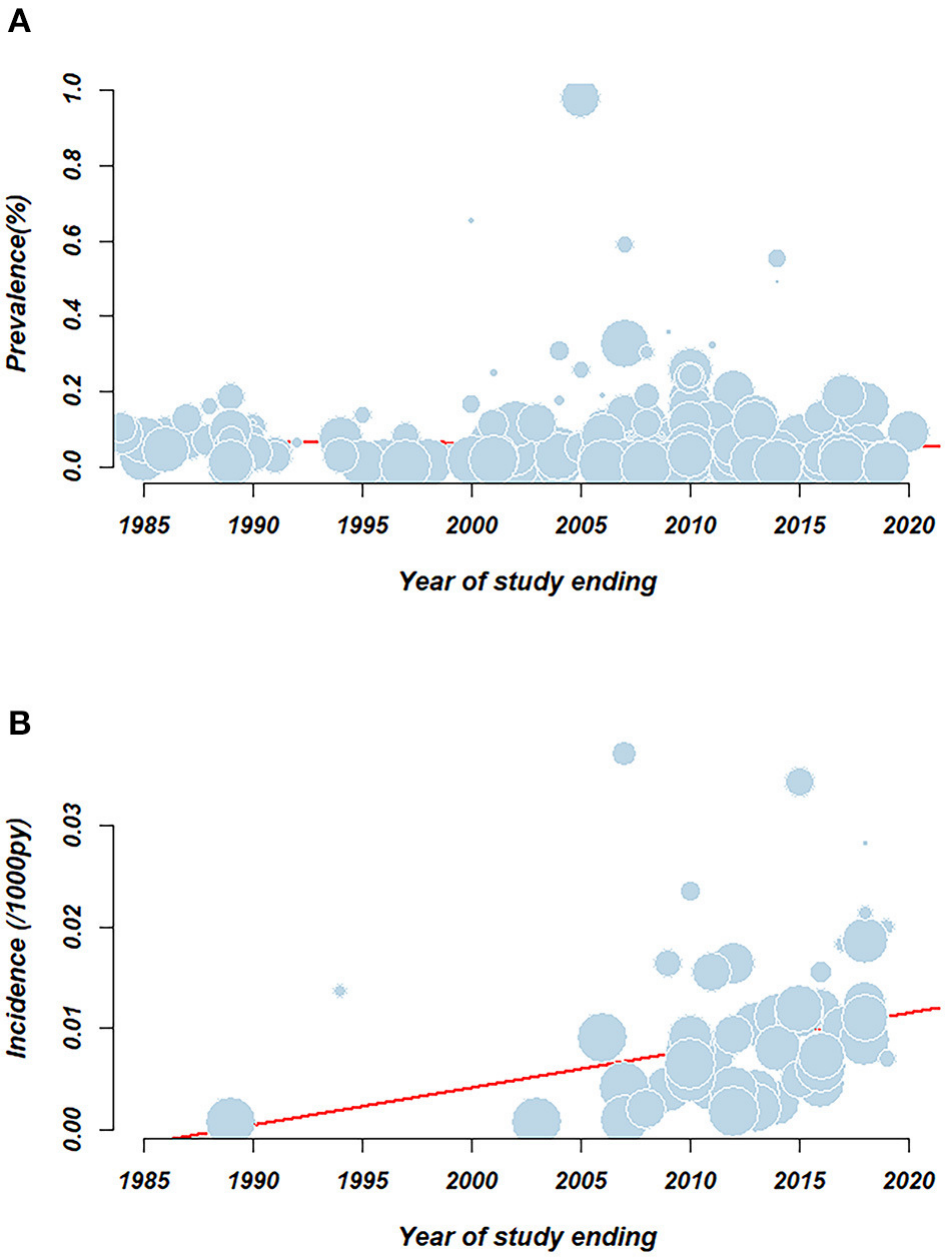
Meta-regression examining the association between pooled rate and study year. **(A)** HCV prevalence; **(B)** HCV incidence. Circles represent individual record. Size of the circle represents the relative weighing of each record. Red line represents the linear regression equation.

To reveal the changing trend of HCV prevalence over time accurately, we also performed a meta-regression to investigate the association between prevalence and the date of study in Figure 3A. No statistically significant association (*p* > 0.05) was demonstrated between the overall HCV prevalence and the year of study end, which indicated that HCV prevalence was relatively stable over 30 years.

### Hepatitis C Virus Prevalence in Participants With Different HIV Infection Statuses

Across 94 records reporting anti-HCV positivity in 54,530 HIV-positive MSM participants, the pooled prevalence of HCV in HIV-positive MSM was 8.1% (95% CI, 6.8–9.4) (*I*^2^ = 97%). As shown in Table 3 and Figure 2B, Indonesia (100.0%, 95% CI: 30.3–100.0), Vietnam (84.1%, 95% CI: 77.7–89.7), Libya (83.3%, 95% CI: 56.1–99.6), Italy (22.6%, 95% CI: 9.1–39.6), and Argentina (21.0%, 95% CI: 8.4–37.3) had a high prevalence exceeding 20%. Japan (2.1%, 95% CI: 1.3–3.0) and Denmark (2.4%, 95% CI: 0.0–7.0) had the lowest prevalence. At the regional level, Asia (7.4%, 95% CI: 3.8–11.7) and Europe (6.3%, 95% CI: 5.1–7.6), particularly Eastern Asia and Northern/Western Europe, had lower HCV burdens than Africa (10.6%, 95% CI: 2.9–21.5), North America (11.4%, 95% CI: 8.9–14.2), Latin America (20.1%, 95% CI: 11.8–29.8), and Oceania (11.6%, 95% CI: 10.6–12.6). High-income countries tended to have the least burden of HCV, with a pooled prevalence of 7.7% (95% CI: 6.5–8.9).

**Table 3:** Estimates of anti-HCV prevalence by HIV status.

**Geographic region**	**HIV+ MSM**			**HIV– MSM**		
	**Record**	**Participant**	**Pooled prevalence estimates, % (95% CI)**	**Record**	**Participant**	**Pooled prevalence estimates, % (95% CI)**
**Asia**	**21**	**9,412**	**7.4 (3.8–11.7)**	**9**	**19,207**	**5.2 (0.8–12.4)**
**Eastern Asia**	**16**	**8,946**	**4.6 (2.9–6.8)**	**7**	**17,757**	**0.7 (0.0–2.1)**
China	7	2,552	5.6 (1.7–11.3)	3	16,635	0.6 (0.5–0.8)
Japan	1	1,068	2.1 (1.3–3.0)			
Mongolia				1	50	36.0 (23.2–49.9)
South Korea	1	320	4.1 (2.1–6.5)			
Taiwan *Province*	7	5,006	4.7 (2.4–7.6)	3	1,072	0.0 (0.0–0.0)
**South-Eastern Asia**	**4**	**366**	**44.9 (0.0–98.8)**	**2**	**1,450**	**24.0 (14.4–35.1)**
Indonesia	1	2	100 (30.3–100.0)	1	75	30.7 (20.7–41.6)
Myanmar	1	177	3.4 (1.1–6.7)			
Thailand	1	42	19.0 (8.4–32.5)			
Vietnam	1	145	84.1 (77.7–89.7)	1	1,375	19.9 (17.9–22.1)
**Western Asia**	**1**	**100**	**3.0 (0.4–7.5)**			
Turkey	1	100	3.0 (0.4–7.5)			
**Europe**	**45**	**26,997**	**6.3 (5.1–7.6)**	**21**	**13,655**	**1.3 (0.7–2.1)**
**Northern Europe**	**9**	**1,935**	**4.5 (1.8–8.1)**	**7**	**2,893**	**0.4 (0.2–0.8)**
Denmark	1	84	2.4 (0.0–7.0)	1	63	0.0 (0.0–0.0)
Sweden	1	59	13.6 (5.8–23.6)	2	1,056	0.1 (0.0–0.6)
United Kingdom	7	1,792	3.9 (1.1–7.9)	4	1,774	0.7 (0.2–1.4)
**Southern Europe**	**8**	**1,278**	**12.7 (5.6–21.9)**	**3**	**895**	**3.6 (1.9–5.6)**
Greece	1	124	8.1 (3.8–13.6)			
Italy	3	200	22.6 (9.1–39.6)	1	519	4.4 (2.8–6.4)
Spain	4	954	8.1 (3.8–13.6)	2	376	3.0 (0.7–6.6)
**Western Europe**	**28**	**23,784**	**5.6 (4.4–7.0)**	**11**	**9,867**	**1.4 (0.6–2.5)**
Belgium	3	509	5.0 (2.4–8.3)	1	132	0.8 (0.0–3.2)
France	7	3,456	4.7 (3.4–6.2)	2	2,278	1.3 (0.0–8.7)
Germany	4	3,084	12.4 (6.5–19.8)	2	3,702	4.1 (0.0–22.7)
Netherlands	7	4,936	5.8 (2.2–10.6)	5	2,934	1.4 (0.4–3.0)
Switzerland	7	11,799	3.8 (2.9–4.8)	1	821	0.4 (0.0–0.9)
**Africa**	**5**	**770**	**10.6 (2.9–21.5)**	**4**	**1,898**	**4.6 (1.9–8.4)**
**Northern Africa**	**1**	**12**	**83.3 (56.1–99.6)**	**1**	**215**	**4.2 (1.9–7.3)**
Libya	1	12	83.3 (56.1–99.6)	1	215	4.2 (1.9–7.3)
**Southern Africa**	**2**	**605**	**4.3 (2.1–7.0)**	**1**	**426**	**2.3 (1.1–4.0)**
South Africa	2	605	4.3 (2.1–7.0)	1	426	2.3 (1.1–4.0)
**Western Africa**	**2**	**153**	**3.6 (0.7–7.8)**	**2**	**1,257**	**6.3 (0.8–16.3)**
Burkina Faso	1	10	10.0 (0.0–38.1)	1	319	11.0 (7.8–14.7)
Nigeria	1	143	4.9 (1.9–9.1)	1	938	3.0 (2.0–4.2)
**Northern America**	**17**	**13,099**	**11.4 (8.9–14.2)**	**12**	**9,411**	**4.4 (2.2–7.2)**
Canada	4	1,409	11.2 (4.7–20.1)	3	746	2.8 (1.6–4.2)
United States	13	11,690	11.5 (8.7–14.7)	9	8,665	4.6 (2.0–8.2)
**Latin**						
**America/Caribbean**	**3**	**246**	**20.1 (11.8–29.8)**	**1**	**410**	**3.2 (1.7–5.1)**
**Central America**	**1**	**61**	**18.0 (9.3–28.8)**			
Mexico	1	61	18.0 (9.3–28.8)			
**South America**	**2**	**185**	**21.0 (8.4–37.3)**	**1**	**410**	**3.2 (1.7–5.1)**
Argentina	2	185	21.0 (8.4–37.3)	1	410	3.2 (1.7–5.1)
**Oceania**	**3**	**4,006**	**11.6 (10.6–12.6)**	**2**	**1,356**	**2.2 (0.1–6.5)**
Australia	3	4,006	11.6 (10.6–12.6)	2	1,356	2.2 (0.1–6.5)
**Global**	**94**	**54,530**	**8.1 (6.8–9.4)**	**49**	**45,937**	**2.8 (1.9–4.0)**

*HCV, hepatitis C virus; CI, confidence interval; HIV, human immunodeficiency virus; MSM, men who have sex with men. Bold values represent results at the global or regional level, and non-bold values represent results at national level*.

A total of 49 studies reported anti-HCV positivity in 45,937 HIV-negative MSM participants. The estimated pooled prevalence was 2.8% (95% CI: 1.9–4.0) (*I*^2^ = 96%), which was substantially lower than that of HIV-positive patients (*p* < 0.001). Table 3 and Figure 2C present a few countries, such as Mongolia (36.0%, 95% CI: 23.2–49.9), Indonesia (30.7%, 95% CI: 20.7–41.6), and Vietnam (19.9%, 95% CI: 17.9–22.1), that were still faced with a high prevalence, while most other countries only had a fairly low prevalence < 3%. Africa (4.6%, 95% CI: 1.9–8.4) and Asia (5.2%, 95% CI: 0.8–12.4) had the highest prevalence, followed by North America (4.4%, 95% CI: 2.2–7.2), Latin America (3.2%, 95% CI: 1.7–5.1), Oceania (2.2%, 95% CI: 0.1–6.5), and Europe (1.3%, 95% CI: 0.7–2.1). A significant difference was found between high-/upper-middle income countries (2.0 and 1.1%) and low-/lower-middle-income countries (19.6 and 11.0%).

### Hepatitis C Virus Incidence and Changing Trend Over Time

A total of 49 cohort studies reported the new development of HCV infection in 704,249 patients with FUPY. The pooled incidence of HCV was 8.6 (95% CI: 7.2–10.0) per 1,000 person-years (*I*^2^ = 96%, Egger's test for bias, *p* = 0.1002). The results of the influential analysis are shown in Supplementary Appendices 3, 4. The omission of an individual study resulted in pooled estimates ranging from 8.2 to 8.8 per 1,000 person-years. Table 1 and Figure 2D present the incidence and FUPY by country. Germany (16.4, 95% CI: 13.3–19.7), Netherlands (11.5, 95% CI: 5.1–20.5), and Australia (11.8, 95% CI: 5.3–21.0) had the highest incidence, while China (2.0, 95% CI: 0.0–8.5), Hong Kong (2.2, 95% CI: 1.2–3.6), and South Korea (2.6, 95% CI: 0.6–5.9) had the lowest incidence. At the regional level, Oceania had the highest incidence, followed by Europe and Northern America, and Asia had the lowest incidence. No data were identified in Africa and Latin America. Almost all the studies were conducted in high-income countries.

Meta-regression demonstrated an association between HCV incidence and the year a study ended (*p* < 0.05) in Figure 3B. There was an increasing trend of incidence over time from 0.8/1,000 person-years (95% CI: 0.1–1.9) in 1989 to 14.1/1,000 person-years (95% CI: 4.6–28.2) in 2021.

## Discussion

This global large-scale meta-analysis provided a comprehensive overview of the HCV prevalence and incidence in the MSM population and the changing trend over time. We indicated that the anti-HCV prevalence is highest in Central America and Southeast Asia in the MSM population. HIV-positive MSM had a much higher HCV prevalence than HIV-negative MSM. An upward trend of incidence was observed globally, especially in developed countries (US, Europe, and Australia), where sexual risk behaviors in MSM were observed increasing substantially (17).

This study found a substantially high HCV prevalence of 5.9% in the MSM population. Compared with the general population, MSM was more prone to conduct high-risk sexual behaviors and had more common HCV infection. According to the WHO data, the estimated HCV prevalence in the general population was 1.5–2.3% in the most affected regions and 0.5–1.0% in the other regions (18). Another global epidemiological systematic review revealed that the average HCV prevalence in the general population was 1.6% (1.3–2.1%) in 2014 (19). However, compared to other high-risk populations, the HCV prevalence identified in MSM was substantially lower. For example, as many as 52.3% (95% CI: 42.4–62.1) of people who inject drugs (PWID) were found to be anti-HCV positive in a global study (20). Another meta-analysis consisted of mainly American, European, and Iranian participants and found that as many as 20.3% (95% CI: 15.5–25.2) of homeless people were HCV infected (21). Data from 46 countries from 2005 to 2015 also revealed that 15.1% of prisoners were anti-HCV positive, with the six WHO regions exceeding 10% (22). The observation that high HCV prevalence in homeless people and prisoners was believed to be highly connected with intravenous drug use, which may largely increase blood-borne virus transmission (21, 22). Although not as severe as PWID, the MSM population still had a considerably higher HCV disease burden compared with the general population, which was non-negligible.

This study also illustrated a wide variation in the prevalence of HCV in MSM among countries and regions. We found that Southeast and Southern Asia, Central America, the Mediterranean, and Eastern Africa had a high prevalence, which was consistent in geographic pattern with that in the general population (19, 23). However, there was still a subtle difference between this study and other studies. In this study, data from the Sub-Saharan population, which was considered a high-prevalence area in general population studies, were not available (24). Notably, Southeast Asia was identified as a high HCV-prevalence area in MSM in this study, but not in the general population (anti-HCV prevalence 1.0%, viremic prevalence 0.7%) (19, 25). The reasons could be multifaceted. First, high rates of drug use that strikingly increased the risk for HCV transmission have been reported among MSM from multiple Southeast Asian countries (26). Second, morbidity of HCV in the MSM population has received less attention in this region, as the 2017 Thai guideline did not recommend testing MSM for HCV on a regular basis (27). Third, the local social culture of conforming to masculine norms and limited acceptance could also put pressure on the MSM population to engage in a series of high-risk behaviors and limited use of harm-reduction services (28). Thus, in addition to conventional strategies of behavioral counseling and health education, regular HCV testing in MSM is emphasized and more efforts are warranted to eliminate sociocultural biases and discrimination toward MSM in Southeast Asia.

This study revealed a particularly high HCV prevalence in HIV-positive MSM. The results aligned with a recent global meta-analysis (29). Both the behavioral and biological factors have been proposed to explain the potential susceptibility of HCV transmission in patients with HIV (30). Biologically, HIV-induced immune impairment might compromise the gastrointestinal mucosa barrier and facilitate HCV transmission (31); additionally, impaired T- and natural killer (NK) cells in patients with HIV could lead to lower rates of HCV seroclearance (32). However, a modeling study indicated that behavioral factors (e.g., sexual behavior risk heterogeneity and HIV preferential mixing) played a pivotal role (30). Multiple studies have shown increased high-risk sexual behavior and decreased precautionary measures among HIV-positive MSM after adopting HIV pre-exposure prophylaxis (PrEP) and a subsequent increase in the incidence of HCV and other sexually transmitted infections (STIs) (33, 34). These findings highlight the urgency of risk-reduction interventions and the importance of routine HCV testing in HIV-positive MSM for all the countries.

A total of 39 studies mainly from Eastern Asia, Europe, North America, and Oceania reported an overall HCV incidence of 8.6/1,000 person-years with an increasing trend over time. Based on this study, developed countries had higher incidences and more significant incidence increases than Asian countries. The findings were consistent with other studies. A European Collaboration reported an increase of HCV incidence from 5.5–8.1 to 23.4–51.1/1,000 person-years during 1995–2007 in 3,014 MSM (35). Another European study also observed a significantly increasing incidence from 0.7 to 18/1,000 person-years during 1990–2014 in 5,941 MSM (36). Similar trend of HCV incidence was also observed in an American MSM cohort, with the lowest of 4.9/1,000 person-years growing to 30.1/1,000 person-years during 2000–2015 (37). In contrast, there was only 2-fold increase of incidence in Asian cohorts (from 2.28 to 4.94/1,000 person-years during 2006–2013 and from 14.28 to 25.38/1,000 person-years during 2011–2018) (38, 39). In fact, HCV incidence in MSM was closely connected with sexual risk behaviors; an increase in these behaviors was proposed as an important reason for increasing incidence (37, 39). Sexual risk behaviors among MSM in developed countries have been higher than in Asian countries including sexualized drug use (40, 41) and condomless anal sex (42, 43). Moreover, sexual risk behaviors of MSM in developed countries are still increasing significantly over time, as seen in the US (44), Denmark (45), UK (46), France (47), and Netherlands (48). The probable causes are condom fatigue, complacency about HIV, availability of other prevention options, optimism about HIV treatments, and the adoption of seroadaptive strategies in the above countries (17). Thus, there is a continued need to reinforce risk-reduction intervention for MSM who engage in unprotected sex and to offer regular HCV monitoring for all the MSM.

Although an increasing HCV incidence was demonstrated by meta-regression, the HCV prevalence was found stable prevalence over time. It was thought to be associated with improved HCV screening and treatment. Notably, HCV treatment had evolved rapidly over the past decades from pegylated interferon in 2000 to the launch of direct-acting antiviral (DAA) drug in 2010 (49). Especially after 2015, the expanded availability of DAA opened a new era of “elimination HCV” (50). In addition to advances in treatment, HCV screening recommendation had also updated and expanded (51). Thus, despite an upward trend of incidence, the increase in cure rate may ensure a relatively stable prevalence.

There are several limitations of this study. First, the current results only report 51 countries globally, with limited generalizability considering the lack of data in many countries. HCV prevalence is higher in low- and middle-income countries, yet reports from these regions (e.g., Sub-Saharan, Eastern Europe, Middle East, North Africa) are inadequate. Thus, more studies from these regions are still needed in these regions. Second, this study has high heterogeneity. Although we explored some sources of heterogeneity such as geographic region, study year, concurrent HIV infection status, income level of countries, controlled the included participants, testing method, outcomes, and study type. However, to comprehensively evaluate the sources of heterogeneity is still limited by the lack of information in most original studies including age distribution, gender, other concurrent high-risk behaviors, or status. Additionally, HCV is less tested among MSM in the early 1990s; furthermore, the sensitivities and specificities of different arrays used in different regions and different time periods for HCV diagnosis may also cause variability. Third, there is a lack of information on some important variables such as PWID. Although it is hindered by the precision in PWID ascertainment in original articles, the PWID status would provide more valuable information as expected. Finally, publication bias was present among studies used to pool the prevalence in MSM population indicating studies with a higher HCV prevalence tend to be more likely to be published. This publication bias may, thus, overestimate the actual HCV prevalence in MSM.

## Conclusion and Implication

This study reveals a consistently higher HCV prevalence in Central America and Southeast Asia in the MSM population. There is also a much higher HCV prevalence in HIV-positive MSM than in HIV-negative MSM. Based on these findings, behavioral counseling, regular HCV testing, and treatment are urgently needed in the above key population and key region. In addition, given the upward trend of HCV incidence and increasing sexual risk behaviors in developed countries, there is a continued need to reinforce risk-reduction intervention for all the MSM.

## Data Availability Statement

The original contributions presented in the study are included in the article/Supplementary Material, further inquiries can be directed to the corresponding author/s.

## Author Contributions

JW, YZhe, and JR designed the study. JW and YZhe developed and implemented the search protocol and wrote the manuscript. YZhe, MY, YZho, YL, JR, and JW abstracted data with JW acting as a tiebreaker at all the stages. YZhe developed the global rates map. JW supervised the whole study and responsible for the decision to submit the manuscript. All authors revised the manuscript from the preliminary draft to submission.

## Funding

This study was supported by grants from the Mega Project of National Science and Technology for the 13th Five-Year Plan of China (2018ZX10721102-003-006 and 2018ZX10715013-003-003) and the National Natural Science Foundation of China (71904170). The funders play no role in the study design, collection, analysis, interpretation of data, the writing of this article, or the decision to submit it for publication.

## Conflict of Interest

The authors declare that the research was conducted in the absence of any commercial or financial relationships that could be construed as a potential conflict of interest.

## Publisher's Note

All claims expressed in this article are solely those of the authors and do not necessarily represent those of their affiliated organizations, or those of the publisher, the editors and the reviewers. Any product that may be evaluated in this article, or claim that may be made by its manufacturer, is not guaranteed or endorsed by the publisher.
