# Supplementary material for: Global Burden and Changing Trend of Hepatitis C Virus Infection in HIV-Positive and HIV-Negative MSM: A Systematic Review and Meta-Analysis
Source: Front Med (Lausanne). 2021 Dec 13;8:774793. doi: 10.3389/fmed.2021.774793 (PMC8710739; doi:10.3389/fmed.2021.774793)
Supplement: Supplementary file 1 [file Data_Sheet_1.DOCX]

**Supplementary materials**

**Appendix 1. Search strategies for database**…………………………………………………2

**Appendix 2. Characteristics and quality assessment of included studies**…………………3

**Appendix 3 Forest plot of influential analysis by excluding each individual study on pooled estimates** ………………………………………………………………………….…18

**Appendix 4. Publication bias and funnel plot**………………………………………………22

**Appendix 5. Forest plot of overall and subgroup prevalence/incidence** …………………24

**Appendix 6. List of included studies**………………………………………………………...29

**Appendix 1. Search strategies for traditional database.**

We conducted a comprehensive search in database of **PubMed, Embase,** and **PsycINFO, CINAHL** using a combination of Medical Subject Headings and free text including terms related to HCV, MSM and prevalence. All related published papers from **January 1, 1990 to July 31, 2021** were identiﬁed and subsequently stored using EndNote X9.

**PubMed**

| **No.** | **Query** | **Results** |
| --- | --- | --- |
| **#1** | Homosexuality, Male [MeSH] OR “men who have sex with men” OR “MSM”OR “homosexual men” OR “gay*” | **38109** |
| **#2** | Hepatitis C [MeSH] OR “ Hepatitis C” OR “HCV” | **100053** |
| **#3** | #1 and #2 | **1025** |

**EMBASE**

| **No.** | **Query** | **Results** |
| --- | --- | --- |
| **#1** | homosexuality OR 'men who have sex with men' OR msm:au OR gay:af | **39261** |
| **#2** | 'hepatitis c virus' OR 'hepatitis c' OR hcv | **179462** |
| **#3** | #1 and #2 | **2071** |

**PsycINFO**

| **No.** | **Query** | **Results** |
| --- | --- | --- |
| **#1** | TX men who have sex with men OR TX MSM OR TX homosexual OR TX gay | **34350** |
| **#2** | TX hepatitis c OR TX HCV | **3807** |
| **#3** | #1 and #2 | **130** |

**Cumulative Index to Nursing and Allied Health Literature (CINAHL)**

| **No.** | **Query** | **Results** |
| --- | --- | --- |
| **#1** | homosexuality or men who have sex with men or msm or gay | **22227** |
| **#2** | hcv or hep c or hepatitis c | **20821** |
| **#3** | #1 and #2 | **377** |

**Appendix 2. Characteristics and quality assessment of included studies**

Published date ranges from 1990 to 2021. Countries with more than 10 included articles were United States (n=30), United Kingdom (n=23), China (n=21), Netherlands (n=14), Spain (n=14), Taiwan province of China (n=12), France (n=11), Canada (n=11), Australia (n=10); countries with 2-10 included articles were Switzerland (n=9), Italy (n=8), Argentina (n=6), Germany (n=5), Belgium (n=4), Denmark (n=3), Sweden (n=3), Tanzania (n=3), Vietnam (n=3), Brazil (n=3), South Africa (n=3), Croatia (n=2), Mexico (n=2), Thailand (n=2), Austria (n=2), Burkina Faso (n=2); remaining 26 countries included only one article. In quality assessment, 107 articles were defined as high quality, while 97 were deemed of medium or low quality. Majority of the cross-sectional studies reported lacks the item of ‘non-respondents’, and most cohort studies lacks ‘non-exposed groups’ and ‘comparability’.

**Supplementary Table 1. Characteristics and quality assessment of prevalence studies**

| Author, Year | Country | UN-DESA region | WB region | Age^#^ | Sample size | HCV testing | Study design | Sampling procedure | Quality assessment |
| --- | --- | --- | --- | --- | --- | --- | --- | --- | --- |
| D'Oliveira Jr, A.,2005 | France | Western Europe | High | / | 1431 | Antibody | Cross-sectional | Convenient | 7 |
| Valadez, J. J.,2013 | Libya | Northern Africa | Upper-mid | / | 224 | Antibody | Cross-sectional | Respondent-driven | 6 |
| Buffington, J.,2007 | US | Northern America | High | / | 1699 | Antibody | Cross-sectional | Convenient | 7 |
| Pando, M. A.,2013 | Argentina | South America | High | 30.5 | 482 | Antibody | Cross-sectional | Respondent-driven | 7 |
| Schmidbauer, C.,2018 | Austria | Western Europe | High | / | 823 | Antibody | Cross-sectional | Convenient | 6 |
| Jiao, Y.,2016 | China | Eastern Asia | Upper-mid | 30.3 | 1296 | Antibody and RNA | Cross-sectional | Convenient | 7 |
| Johns, D. G.,1998 | Canada | Northern America | High | / | 167 | Antibody | Cross-sectional | Convenient | 5 |
| Deiss, R. G.,2008 | Mexico | Central America | Upper-mid | 34 | 115 | Antibody | Cross-sectional | Respondent-driven | 7 |
| Tor, J.,1990 | Spain | Southern Europe | High | 34.2 | 105 | Antibody | Cross-sectional | Convenient | 6 |
| Fainboim, H.,1999 | Argentina | South America | High | 29 | 99 | Antibody | Cross-sectional | Convenient | 5 |
| Price, H.,2013 | UK | Northern Europe | High | 33 | 1121 | Antibody | Cross-sectional | Convenient | 7 |
| Lee, S.,2016 | South Korea | Eastern Asia | High | 44 | 320 | Antibody | Cohort (baseline) | Convenient | 7 |
| Wandeler, G.,2012 | Switzerland | Western Europe | High | 38 | 4629 | Antibody | Cohort (baseline) | Convenient | 8 |
| van de Laar, T. J.,2007 | Netherlands | Western Europe | High | 31.8 | 1836 | Antibody | Cohort (baseline) | Convenient | 9 |
| Rivas-Estilla, A. M.,2007 | Mexico | Central America | Upper-mid | 34 | 62 | Antibody | Cross-sectional | Convenient | 6 |
| Seaberg, E. C.,2014 | US | Northern America | High | 33.5 | 6890 | Antibody | Cross-sectional | Cluster | 7 |
| Ruan, Y.,2009 | China | Eastern Asia | Upper-mid | 27 | 541 | Antibody | Cross-sectional | Convenient | 6 |
| Qin, Q.,2016 | China | Eastern Asia | Upper-mid | / | 129136 | Antibody | Cross-sectional | Stratified+Snowball | 6 |
| Morano, J. P.,2013 | US | Northern America | High | / | 189 | Antibody | Cross-sectional | Convenient | 6 |
| Ireland, G.,2017 | UK | Northern Europe | High | 33 | 2030 | Antibody | Cross-sectional | Convenient | 7 |
| Mmbaga, E. J.,2017 | Tanzania | Eastern Africa | Low | 27 | 409 | Antibody | Cross-sectional | Respondent-driven | 7 |
| Andreu, J.,1994 | Spain | Southern Europe | High | 29.6 | 168 | Antibody | Cross-sectional | Convenient | 5 |
| Saillour, F.,1996 | France | Western Europe | High | / | 667 | Antibody | Cross-sectional | Convenient | 6 |
| Gogela, N. A.,2018 | South Africa | Southern Africa | Upper-mid | 36 | 285 | Antibody | Cross-sectional | Convenient | 7 |
| Rauch, A.,2005 | Switzerland | Western Europe | High | 37 | 2550 | Antibody | Cohort (baseline) | Convenient | 9 |
| Baral, S.,2010 | Russia | Eastern Europe | Upper-mid | / | 50 | Antibody | Cohort (baseline) | Convenient+Snowball | 6 |
| Lee, S. D.,1991 | Taiwan | Eastern Asia | High | 33 | 26 | Antibody | Cross-sectional | Convenient | 4 |
| Wong, J.,2015 | Canada | Northern America | High | 33 | 900 | Antibody | Cross-sectional | Convenient | 6 |
| Van der Poel, C. L.,1991 | Netherlands | Western Europe | High | / | 225 | Antibody | Cross-sectional | Convenient | 7 |
| Berini, C. A.,2007 | Argentina | South America | High | / | 682 | Antibody | Cross-sectional | Convenient | 7 |
| Tieu, H. V.,2018 | US | Northern America | High | / | 1028 | Antibody | Cross-sectional | Convenient | 8 |
| Marongiu, A.,2012 | UK | Northern Europe | High | 29 | 8671 | Antibody | Cross-sectional | Convenient | 6 |
| Ouedraogo, H. G.,2018 | Burkina Faso | Western Africa | Low | 22.9 | 329 | Antibody | Cross-sectional | Respondent-driven | 7 |
| Jansen, K.,2015 | Germany | Western Europe | High | / | 1838 | Antibody | Cohort (baseline) | Convenient | 6 |
| Sun, H. Y.,2014 | Taiwan | Eastern Asia | High | / | 1128 | Antibody | Cohort (baseline) | Convenient | 9 |
| Kouyos, R. D.,2014 | Switzerland | Western Europe | High | / | 3730 | Antibody | Cohort (baseline) | Convenient | 8 |
| Ronn, M.,2014 | UK | Northern Europe | High | / | 1020 | Antibody | Cross-sectional | Convenient | 6 |
| Ward, C.,2014 | UK | Northern Europe | High | 34 | 471 | Antibody | Cross-sectional | Convenient | 6 |
| Mayer, K. H.,2012 | US | Northern America | High | / | 1155 | Antibody | Cross-sectional | Convenient | 6 |
| Ndimbie, O. K.,1995 | US | Northern America | High | / | 617 | Antibody | Cohort (baseline) | Convenient | 6 |
| Ndimbie, O. K.,1996 | US | Northern America | High | / | 1051 | Antibody | Cross-sectional | Convenient | 7 |
| Yehia, B. R.,2014 | US | Northern America | High | / | 3273 | Antibody | Cross-sectional | Convenient | 6 |
| Jin, F.,2005 | Australia | Australia/New Zealand | High | 35 | 824 | Antibody | Cohort (baseline) | Convenient | 6 |
| Tedaldi, E. M.,2003 | US | Northern America | High | 39.1 | 2182 | Antibody | Cohort (baseline) | Convenient | 6 |
| Melbye, M.,1990 | Denmark | Northern Europe | High | / | 250 | Antibody | Cohort (baseline) | Convenient | 6 |
| Vaux, S.,2019 | France | Western Europe | High | 41 | 2645 | Antibody and RNA | Cross-sectional | Convenient | 8 |
| Hopwood, M.,2016 | Australia | Australia/New Zealand | High | 44.4 | 474 | Antibody | Cross-sectional | Convenient | 4 |
| Parisi, S. G.,2011 | Italy | Southern Europe | High | 42 | 133 | Antibody | Cohort (baseline) | Convenient | 6 |
| Hao, C.,2011 | China | Eastern Asia | Upper-mid | / | 416 | Antibody | Cohort (baseline) | Respondent-driven | 9 |
| Mohsen, A. H.,2005 | UK | Northern Europe | High | / | 375 | Antibody | Cross-sectional | Convenient | 7 |
| Tedder, R. S.,1991 | UK | Northern Europe | High | / | 275 | Antibody | Cross-sectional | Convenient | 8 |
| Schmidt, A. J.,2011 | Germany | Western Europe | High | / | 4385 | Antibody | Cross-sectional | Convenient | 6 |
| Cavlek, T. V.,2009 | Croatia | Southern Europe | High | / | 205 | Antibody | Cross-sectional | Convenient | 7 |
| Hu, J.,2017 | China | Eastern Asia | Upper-mid | / | 15705 | Antibody | Cross-sectional | Convenient | 7 |
| Serrano-Villar, S.,2015 | Spain | Southern Europe | High | / | 3722 | Antibody | Cross-sectional | Convenient | 9 |
| Sprenger, K.,2014 | Switzerland | Western Europe | High | / | 112 | Antibody | Cross-sectional | Convenient | 5 |
| Cotte, L.,2018 | France | Western Europe | High | 41 | 10049 | Antibody | Cohort (baseline) | Convenient | 9 |
| Bodsworth, N. J.,1996 | Australia | Australia/New Zealand | High | 36 | 1038 | Antibody | Cross-sectional | Convenient | 7 |
| Shen, L.,2017 | China | Eastern Asia | Upper-mid | / | 657 | Antibody | Cross-sectional | Convenient | 7 |
| Buxton, J. A.,2010 | Canada | Northern America | High | / | 843 | Antibody | Cross-sectional | Convenient | 7 |
| Ward, H.,2007 | UK | Northern Europe | High | / | 251 | Antibody | Cross-sectional | Convenient | 6 |
| Matser, A.,2013 | Netherlands | Western Europe | High | 43 | 786 | Antibody | Cross-sectional | Convenient | 7 |
| Li, C. W.,2018 | Taiwan | Eastern Asia | High | / | 5170 | Antibody | Cross-sectional | Convenient | 7 |
| Jebbari, H.,2007 | UK | Northern Europe | High | / | 411 | Antibody | Cross-sectional | Convenient | 5 |
| Lincoln, D.,2003 | Australia | Australia/New Zealand | High | / | 1338 | Antibody | Cohort (baseline) | Cluster | 6 |
| Chalmet, K.,2010 | Belgium | Western Europe | High | / | 237 | Antibody | Cohort (baseline) | Convenient | 7 |
| Pereira, G. A. S.,2006 | Brazil | South America | Upper-mid | / | 37 | Antibody | Cross-sectional | Convenient | 7 |
| Pando, M. A.,2012 | Argentina | South America | High | 30.5 | 496 | Antibody | Cross-sectional | Respondent-driven | 7 |
| Vicknasingam, Balasingam,2009 | Malaysia | South-Eastern Asia | Upper-mid | 37 | 179 | Antibody | Cross-sectional | Convenient | 5 |
| Kouyos, R. D.,2014 | Switzerland | Western Europe | High | 34 | 671 | Antibody | Cohort (baseline) | Convenient | 8 |
| Marcellin, P.,1993 | France | Western Europe | High | 34 | 113 | Antibody | Cross-sectional | Convenient | 5 |
| Gao, W.,2015 | China | Eastern Asia | Upper-mid | 30 | 600 | Antibody | Cross-sectional | Snowball | 7 |
| Anand, C. M.,1992 | Canada | Northern America | High | / | 101 | Antibody | Cross-sectional | Convenient | 4 |
| Colon-Lopez, V.,2011 | Puerto Rico | Caribbean | High | 43.4 | 41 | Antibody | Cross-sectional | Convenient | 6 |
| Schmidt, A. J.,2014 | Switzerland | Western Europe | High | 33 | 840 | Antibody | Cross-sectional | Convenient | 5 |
| Balogun, M. A.,2003 | UK | Northern Europe | High | / | 2599 | Antibody | Cross-sectional | Convenient | 7 |
| Alary, M.,2005 | Canada | Northern America | High | 32 | 1085 | Antibody | Cohort (baseline) | Convenient | 6 |
| Garg, S.,2013 | US | Northern America | High | / | 1059 | Antibody | Cohort (baseline) | Convenient | 9 |
| Nadol, P.,2016 | Vietnam | South-Eastern Asia | Lower-mid | 26.8 | 1520 | Antigen/Antibody | Cross-sectional | Respondent-driven | 7 |
| Remis, R. S.,2016 | Canada | Northern America | High | 44.4 | 437 | Antibody | Cross-sectional | Convenient | 6 |
| Braun, D. L.,2019 | Switzerland | Western Europe | High | 46.7 | 3722 | Antibody | Cohort (baseline) | Convenient | 5 |
| Ghosn, J.,2006 | France | Western Europe | High | 34 | 252 | Antibody | Cohort (baseline) | Convenient | 6 |
| Daskalopoulou, M.,2014 | UK | Northern Europe | High | / | 1216 | Antibody or RNA | Cross-sectional | Convenient | 7 |
| Hughes, G.,2013 | UK | Northern Europe | High | / | 1088 | Antibody | Cross-sectional | Convenient | 6 |
| He, Q.,2009 | China | Eastern Asia | Upper-mid | / | 423 | Antibody | Cross-sectional | Respondent-driven | 7 |
| Cohen, D. E.,2006 | US | Northern America | High | 36 | 218 | Antibody | Cross-sectional | Convenient | 6 |
| Quaranta, J. F.,1994 | France | Western Europe | High | 30.9 | 68 | Antibody | Cohort (baseline) | Convenient | 7 |
| Pasvol, T.,2016 | UK | Northern Europe | High | / | 794 | Antibody | Cross-sectional | Convenient | 6 |
| Sun, H. Y.,2012 | Taiwan | Eastern Asia | High | 43 | 731 | Antibody | Case-control | Convenient | 8 |
| Corona, R.,1991 | Italy | Southern Europe | High | 32 | 195 | Antibody | Cross-sectional | Convenient | 7 |
| Soares, C. C.,2014 | Brazil | South America | Upper-mid | 23 | 558 | Antibody | Cross-sectional | Respondent-driven | 7 |
| Sirinak, C.,2008 | Thailand | South-Eastern Asia | Upper-mid | 34 | 42 | Antibody | Cohort (baseline) | Convenient | 6 |
| Herrera, M. C.,2015 | Peru | South America | Upper-mid | 30 | 399 | Antibody | Cross-sectional | Convenient | 6 |
| Jansen, K.,2013 | Germany | Western Europe | High | 33 | 1945 | Antibody | Cross-sectional | Convenient | 6 |
| Adebajo, Sylvia Bolanle,2014 | Nigeria | Western Africa | Lower-mid | 23.7 | 1125 | Antibody | Cross-sectional | Respondent-driven | 9 |
| Alfonso Gil, R.,1999 | Spain | Southern Europe | High | / | 199 | Antibody | Cross-sectional | Convenient | 7 |
| An, M. H.,2013 | China | Eastern Asia | Upper-mid | 31.5 | 513 | Antibody | Cross-sectional | Convenient | 7 |
| Beyrer, C.,2005 | Thailand | South-Eastern Asia | Upper-mid | / | 66 | Antibody | Cross-sectional | Convenient | 6 |
| Blaxhult, A.,2014 | Sweden | Northern Europe | High | 33 | 1008 | Antibody | Cross-sectional | Convenient | 6 |
| Bollepalli, S.,2007 | US | Northern America | High | / | 142 | Antibody | Cross-sectional | Convenient | 7 |
| Bozicevic, I.,2009 | Croatia | Southern Europe | High | 27 | 360 | Antibody | Cross-sectional | Respondent-driven | 6 |
| Buchbinder, S. P.,1994 | US | Northern America | High | 34 | 435 | Antibody | Cohort (baseline) | Convenient | 4 |
| Carlos Martin, J.,2004 | Spain | Southern Europe | High | / | 119 | Antibody | Cross-sectional | Convenient | 7 |
| Clerc, O.,2016 | Switzerland | Western Europe | High | 35 | 512 | Antibody | Cross-sectional | Convenient | 7 |
| Colby, D.,2012 | Vietnam | South-Eastern Asia | Lower-mid | 21 | 300 | Antibody | Cross-sectional | Convenient | 6 |
| Coll, J.,2018 | Spain | Southern Europe | High | 34.2 | 254 | Antibody | Cohort (baseline) | Convenient | 6 |
| Cuomo, G.,2018 | Italy | Southern Europe | High | 47 | 442 | Antibody | Cohort (baseline) | Convenient | 9 |
| Dahoma, M.,2011 | Tanzania | Eastern Africa | Low | 28 | 509 | Antibody | Cross-sectional | Respondent-driven | 7 |
| Davaalkham, J.,2009 | Mongolia | Eastern Asia | Lower-mid | 28.5 | 50 | Antibody | Cross-sectional | Cluster | 5 |
| Defraye, A.,2011 | Belgium | Western Europe | High | 39.5 | 241 | Antibody | Cross-sectional | Convenient | 7 |
| Diamond, C.,2003 | US | Northern America | High | / | 824 | Antibody | Cross-sectional | Convenient | 6 |
| Dimitrakopoulos, A.,2000 | Greece | Southern Europe | High | / | 124 | Antibody | Cohort (baseline) | Convenient | 5 |
| Duong, H. T.,2018 | Vietnam | South-Eastern Asia | Lower-mid | 32 | 45 | Antibody | Cross-sectional | Respondent-driven | 5 |
| El-Hayek, C.,2014 | Australia | Australia/New Zealand | High | / | 2190 | Antibody or RNA | Cohort (baseline) | Convenient | 6 |
| Filippini, P.,2003 | Italy | Southern Europe | High | 32 | 67 | Antibody | Cross-sectional | Convenient | 5 |
| Fiscus, S. A.,1994 | US | Northern America | High | / | 182 | Antibody | Cross-sectional | Convenient | 5 |
| Forbes, K. M.,2009 | UK | Northern Europe | High | 41 | 117 | Antibody | Cross-sectional | Convenient | 6 |
| Francisci, D.,1995 | Italy | Southern Europe | High | / | 30 | Antibody | Cross-sectional | Convenient | 5 |
| Gasparini, V.,1991 | Italy | Southern Europe | High | / | 337 | Antibody | Cross-sectional | Convenient | 7 |
| Goldberg, D.,2001 | UK | Northern Europe | High | / | 668 | Antibody | Cross-sectional | Convenient | 6 |
| Golden, M. R.,2006 | US | Northern America | High | / | 198 | Antibody | Cross-sectional | Snowball | 7 |
| Guo, H.,2009 | China | Eastern Asia | Upper-mid | / | 433 | Antibody | Cross-sectional | Convenient | 7 |
| Hammer, G. P.,2003 | US | Northern America | High | / | 746 | Antibody | Cohort (baseline) | Convenient | 6 |
| He, Q.,2006 | China | Eastern Asia | Upper-mid | 29.5 | 177 | Antibody | Cross-sectional | Convenient | 6 |
| Heiligenberg, M.,2012 | Netherlands | Western Europe | High | 46 | 649 | Antibody | Cross-sectional | Convenient | 7 |
| Ho, S. Y.,2017 | Taiwan | Eastern Asia | High | / | 2337 | Antibody | Cohort (baseline) | Convenient | 6 |
| Hoornenborg, E.,2017 | Netherlands | Western Europe | High | / | 375 | Antibody or RNA | Cohort (baseline) | Convenient | 6 |
| Johnston, L. G.,2010 | Tanzania | Eastern Africa | Low | / | 509 | Antibody | Cross-sectional | Respondent-driven | 7 |
| Johnston, L. G.,2013 | Dominican Republic | Caribbean | Upper-mid | 23 | 1388 | Antibody | Cross-sectional | Respondent-driven | 7 |
| Assi, A.,2018 | Lebanon | Western Asia | Upper-mid | / | 1351 | Antibody | Cross-sectional | Convenient | 5 |
| Khanani, M. R.,2010 | Pakistan | Southern Asia | Lower-mid | / | 396 | Antibody | Cross-sectional | Respondent-driven | 7 |
| Kiese, M.,1990 | Germany | Western Europe | High | / | 267 | Antibody | Cross-sectional | Convenient | 5 |
| Ko, N. Y.,2006 | Taiwan | Eastern Asia | High | / | 325 | Antibody | Cross-sectional | Convenient | 8 |
| Ko, N. Y.,2011 | Taiwan | Eastern Asia | High | 34.2 | 1002 | Antibody | Cross-sectional | Convenient | 8 |
| Lachowsky, N. J.,2016 | Canada | Northern America | High | / | 774 | Antibody | Cohort (baseline) | Respondent-driven | 6 |
| Larsen, C.,2008 | France | Western Europe | High | / | 558 | Antibody | Cross-sectional | Stratified | 7 |
| Lee, H. C.,2008 | Taiwan | Eastern Asia | High | 30.9 | 113 | Antibody | Cross-sectional | Convenient | 9 |
| Lissen, E.,1993 | Spain | Southern Europe | High | 29.6 | 168 | Antibody | Cross-sectional | Convenient | 5 |
| Liu, J.,2015 | China | Eastern Asia | Upper-mid | / | 963 | Antibody | Cross-sectional | Convenient | 7 |
| Lu, S.,2017 | China | Eastern Asia | Upper-mid | 32.7 | 102 | Antibody | Cross-sectional | Snowball | 5 |
| Ma, X.,2007 | China | Eastern Asia | Upper-mid | / | 1292 | Antibody | Cross-sectional | Respondent-driven | 7 |
| Mikati, T.,2018 | US | Northern America | High | 28 | 1139 | Antibody | Cross-sectional | Convenient | 5 |
| Nasir, A.,2011 | Afghanistan | Southern Asia | Low | / | 124 | Antibody | Cross-sectional | Convenient | 6 |
| Newsum, A. M.,2018 | Netherlands | Western Europe | High | 32 | 504 | Antibody | Cross-sectional | Convenient | 7 |
| Nishijima, T.,2014 | Japan | Eastern Asia | High | / | 1068 | Antibody | Cohort (baseline) | Convenient | 7 |
| Opravil, M.,1998 | Switzerland | Western Europe | High | / | 96 | Antibody | Cross-sectional | Convenient | 9 |
| Osella, A. R.,1998 | Argentina | South America | High | / | 228 | Antibody | Cross-sectional | Convenient | 6 |
| Osmond, D. H.,1993 | US | Northern America | High | 35 | 735 | Antibody | Cross-sectional | Convenient | 7 |
| Oster, A. M.,2014 | US | Northern America | High | / | 438 | Antibody | Cross-sectional | Stratified | 7 |
| Palacios, R.,2009 | Spain | Southern Europe | High | 44.4 | 727 | Antibody | Cross-sectional | Convenient | 7 |
| Pallawela, S.,2010 | UK | Northern Europe | High | 36 | 226 | Antibody | Cross-sectional | Convenient | 5 |
| Pando, M. A.,2006 | Argentina | South America | High | 30.9 | 681 | Antibody | Cross-sectional | Convenient | 7 |
| Prasetyo, A. A.,2014 | Indonesia | South-Eastern Asia | Lower-mid | 27 | 77 | Antibody | Cross-sectional | Respondent-driven | 6 |
| Price, H.,2017 | UK | Northern Europe | High | / | 294 | Antibody | Cross-sectional | Convenient | 5 |
| Raymond, H. F.,2012 | US | Northern America | High | / | 466 | Antibody | Cross-sectional | Convenient | 7 |
| Raymond, H. F.,2011 | US | Northern America | High | / | 207 | Antibody | Cross-sectional | Convenient | 7 |
| Ricchi, E.,1992 | Italy | Southern Europe | High | 32 | 622 | Antibody | Cross-sectional | Convenient | 7 |
| Roca, B.,2003 | Spain | Southern Europe | High | 37 | 510 | Antibody | Cross-sectional | Convenient | 6 |
| Ruutel, K.,2015 | Estonia | Northern Europe | High | / | 43 | Antibody | Cross-sectional | Convenient | 5 |
| Sanchez-Quijano, A.,1990 | Spain | Southern Europe | High | 28 | 146 | Antibody | Cross-sectional | Convenient | 4 |
| Scott, C.,2010 | UK | Northern Europe | High | 33 | 2309 | Antibody | Cross-sectional | Convenient | 6 |
| Sonderup, M. W.,2017 | South Africa | Southern Africa | Upper-mid | 34 | 541 | Antibody | Cross-sectional | Convenient | 7 |
| Sonnerborg, A.,1990 | Sweden | Northern Europe | High | / | 107 | Antibody | Cross-sectional | Convenient | 5 |
| Spinner, C. D.,2018 | Germany | Western Europe | High | 43.2 | 296 | Antibody | Cross-sectional | Convenient | 5 |
| Tseng, Y. T.,2012 | Taiwan | Eastern Asia | High | / | 1123 | Antibody | Cross-sectional | Convenient | 7 |
| Urbanus, A. T.,2014 | Netherlands | Western Europe | High | 40 | 777 | Antibody | Cross-sectional | Convenient | 7 |
| van Rooijen, M.,2016 | Netherlands | Western Europe | High | 41 | 2156 | Antibody | Cohort (baseline) | Convenient | 9 |
| Vanderschueren, S.,1991 | Belgium | Western Europe | High | / | 163 | Antibody | Cross-sectional | Convenient | 7 |
| Wang, L.,2013 | China | Eastern Asia | Upper-mid | 29.4 | 125528 | Antibody | Cross-sectional | Convenient | 7 |
| Westh, H.,1993 | Denmark | Northern Europe | High | 34 | 147 | Antibody | Cross-sectional | Convenient | 4 |
| Widell, A.,1991 | Sweden | Northern Europe | High | / | 211 | Antibody | Cross-sectional | Convenient | 6 |
| Wilkinson, F.,2010 | UK | Northern Europe | High | / | 182 | Antibody | Cross-sectional | Convenient | 6 |
| Zaw, S. K.,2013 | Myanmar | South-Eastern Asia | Lower-mid | 36 | 177 | Antibody | Cross-sectional | Convenient | 6 |
| Zhang, X.,2007 | China | Eastern Asia | Upper-mid | 26.1 | 753 | Antibody | Cross-sectional | Convenient | 6 |
| Zhao, Y. S.,2012 | China | Eastern Asia | Upper-mid | 31.4 | 230 | Antibody | Cross-sectional | Convenient | 6 |
| Zhou, L.,2015 | China | Eastern Asia | Upper-mid | 38.5 | 381 | Antibody | Cross-sectional | Convenient | 5 |
| Zhou, Z. H.,2010 | China | Eastern Asia | Upper-mid | 27.5 | 550 | Antibody | Cross-sectional | Convenient+Snowball | 9 |
| Zohrabyan, L.,2013 | Moldova | Eastern Europe | Lower-mid | 20 | 391 | Antibody | Cross-sectional | Respondent-driven | 7 |
| Cardona-Arias, J. A.,2020 | Colombia | South America | Upper-middle |  | 1100 | Antibody | Cross-sectional | Convenient | 9 |
| Chen, Y. C.,2020 | US | Northern America | High | 40.9 | 1289 | Antibody or RNA | Cross-sectional | Convenient | 9 |
| Clipman, S. J.,2020 | India | Southern Asia | Lower-middle | 25 | 4994 | Antibody | Cross-sectional | Respondent-driven | 9 |
| Davison, K. L.,2021 | UK | Northern Europe | High |  | 115 | Antibody | Cross-sectional | Convenient | 7 |
| de Andrade, A..A.,2019 | Brazil | South America | Upper-middle |  | 522 | Antibody | Cross-sectional | Respondent-driven | 5 |
| Irvin, R.,2020 | US | Northern America | High | 41 | 1287 | Antibody | Cross-sectional | Respondent-driven | 9 |
| Jongen, V. W.,2020 | Netherlands | Western Europe | High |  | 1003 | Antibody | Cross-sectional | Convenient | 9 |
| Lanièce Delaunay, C.,2021 | Canada | Northern America | High |  | 1086 | Antibody | Cross-sectional | Convenient | 9 |
| Lee, C. Y.,2021 | Taiwan | Eastern Asia | High |  | 620 | Antibody | Cross-sectional | Convenient | 9 |
| Lu, R.,2020 | China | Eastern Asia | Upper-middle |  | 4900 | Antibody | Cross-sectional | Convenient | 8 |
| Sahin, M.,2021 | Turkey | Western Asia | Upper-middle |  | 100 | Antibody | Cross-sectional | Convenient | 6 |
| Scheibe, A.,2020 | South Africa | Southern Africa | Upper-middle |  | 746 | Antibody | Cross-sectional | Convenient | 9 |
| Scheim, A.,2019 | Canada | Northern America | High |  | 225 | Antibody | Cohort (baseline) | Convenient | 6 |
| Willekens, R.,2021 | Spain | Southern Europe | High |  | 301 | RNA | Cross-sectional | Convenient | 4 |
| Yaya, I.,2021 | Mali | Western Africa | Low |  | 320 | Antibody | Cross-sectional | Convenient | 8 |
| Yaya, I.,2021 | Cote d’Ivoire | Western Africa | Lower-middle |  | 206 | Antibody | Cross-sectional | Convenient | 8 |
| Yaya, I.,2021 | Burkina Faso | Western Africa | Low |  | 183 | Antibody | Cross-sectional | Convenient | 8 |
| Yaya, I.,2021 | Togo | Western Africa | Low |  | 171 | Antibody | Cross-sectional | Convenient | 8 |
| ^#^Data of Age are presented as median or mean. Adapted Newcastle Ottawa Scale (NOS) is used in quality assessment for Cross-sectional, Cohort, and Case-control, studies with scores of 7-9 are considered high quality, 4-6 are medium quality, 0-3 are low quality. | | | | | | | | | |

**Supplementary Table 2. Characteristics and quality assessment of incidence studies**

| Author, Year | Country | UN-DESA region | WB region | Age^#^ | FUPY | HCV testing | Study design | Sampling method | Quality assessment |
| --- | --- | --- | --- | --- | --- | --- | --- | --- | --- |
| Giraudon, I.,2008 | UK | Northern Europe | High | / | 42985 | Antibody or RNA | Cohort | Cluster | 6 |
| Samandari, T.,2017 | US | Northern America | High | / | 5829 | Antibody | Cohort | Cluster | 9 |
| Boerekamps, A.,2018 | Netherlands | Western Europe | High | 42 | 8290 | RNA | Cohort | Convenient | 6 |
| Boerekamps, A.,2018 | Netherlands | Western Europe | High | 46 | 8961 | RNA | Cohort | Convenient | 6 |
| Medland, N. A.,2017 | Australia | Australia/New Zealand | High | 43.1 | 3114 | Antibody or RNA | Cohort | Convenient | 6 |
| Boettiger, D. C.,2017 | Australia | Australia/New Zealand | High | 41 | 3358 | Antibody or RNA | Cohort | Convenient | 9 |
| Hoornenborg, E.,2018 | Netherlands | Western Europe | High | / | 658 | Antibody | Cohort | Convenient | 6 |
| Lee, S.,2016 | South Korea | Eastern Asia | High | / | 1550 | Antibody | Cohort | Convenient | 7 |
| Wandeler, G.,2012 | Switzerland | Western Europe | High | / | 23707 | Antibody | Cohort | Convenient | 8 |
| Sobrino-Vegas, P.,2014 | Spain | Southern Europe | High | / | 3615 | Antibody | Cohort | Convenient | 8 |
| van de Laar, T. J.,2007 | Netherlands | Western Europe | High | 31.8 | 12216 | Antibody | Cohort | Convenient | 9 |
| Chaillon, A.,2019 | US | Northern America | High | 39 | 15796 | Antibody or RNA | Cohort | Convenient | 9 |
| Tsai, J. C.,2015 | Taiwan | Eastern Asia | High | 29 | 3678 | Antibody | Cohort | Convenient | 9 |
| Barfod, T. S.,2011 | Denmark | Northern Europe | High | 41 | 3484 | Antibody and RNA | Cohort | Convenient | 6 |
| Jansen, K.,2015 | Germany | Western Europe | High | / | 6054 | Antibody | Cohort | Convenient | 6 |
| Donahue, J. G.,1991 | US | Northern America | High | / | 3927 | Antibody | Cohort | Convenient | 7 |
| Larsen, C.,2011 | France | Western Europe | High | / | 24445 | Antibody | Cohort | Convenient | 6 |
| Chen, Y. C.,2016 | US | Northern America | High | 38.4 | 5242 | Antibody | Cohort | Convenient | 9 |
| Brook, G.,2013 | UK | Northern Europe | High | / | 353 | Antibody | Cohort | Convenient | 9 |
| Lin, A. W.,2014 | Hong Kong | Eastern Asia | High | 38 | 6295 | Antibody | Cohort | Convenient | 6 |
| Rockstroh, J.,2012 | Europe | / | / | / | 18928 | Antibody | Cohort | Convenient | 6 |
| Cotte, L.,2018 | France | Western Europe | High | / | 4151 | Antibody | Cohort | Convenient | 9 |
| Gamage, D. G.,2011 | Australia | Australia/New Zealand | High | / | 4359 | Antibody | Cohort | Convenient | 7 |
| Sanchez, C.,2013 | Spain | Southern Europe | High | / | 4982 | Antibody and RNA | Cohort | Convenient | 6 |
| Jin, F.,2010 | Australia | Australia/New Zealand | High | / | 4650 | / | Cohort | Convenient | 9 |
| Garg, S.,2013 | US | Northern America | High | / | 1408 | Antibody | Cohort | Convenient | 9 |
| Burchell, A. N.,2015 | Canada | Northern America | High | 41 | 9987 | Antibody | Cohort | Convenient | 9 |
| Breskin, A.,2015 | US | Northern America | High | 34 | 315392 | Antibody or RNA | Cohort | Convenient | 9 |
| Chaillon, A.,2017 | US | Northern America | High | 38 | 12573 | Antibody or RNA | Cohort | Convenient | 8 |
| Cuomo, G.,2018 | Italy | Southern Europe | High | / | 4335 | Antibody | Cohort | Convenient | 9 |
| El-Hayek, C.,2014 | Australia | Australia/New Zealand | High | / | 4065 | Antibody or RNA | Cohort | Convenient | 6 |
| Giuliani, M.,1997 | Italy | Southern Europe | High | 35 | 365 | Antibody | Cohort | Convenient | 6 |
| Lachowsky, N. J.,2016 | Canada | Northern America | High | / | 1000 | Antibody | Cohort | Repondent-driven | 6 |
| Nishijima, T.,2014 | Japan | Eastern Asia | High | 25 | 2246 | Antibody | Cohort | Convenient | 7 |
| Pradat, P.,2018 | France | Western Europe | High | 45 | 30231 | Antibody or RNA | Cohort | Cluster | 7 |
| Ruan, Y.,2009 | China | Eastern Asia | Upper-mid | 26 | 507 | Antibody | Cohort | Convenient | 6 |
| van der Helm, J. J.,2011 | Netherlands | Western Europe | High | / | 2488 | Antibody or RNA | Cohort | Convenient | 7 |
| van Rooijen, M.,2016 | Netherlands | Western Europe | High | / | 1234 | Antibody | Cohort | Convenient | 9 |
| Vanhommerig, J. | Netherlands | Western Europe | High | 30.3 | 17310 | Antibody | Cohort | Convenient | 6 |
| Ang, L. W.,2021 | Singapore | Southeast Asia | High | 43 | 4012 | Antibody | Cohort | Clusting | 9 |
| Garvey, L. J.,2020 | UK | Northern Europe | High |  | 42899 | Antibody or RNA | Cohort | Convenient | 8 |
| Gonzalez-Serna, A.,2021 | Spain | Southern Europe | High | 44.7 | 704 | Antibody | Cohort | Convenient | 9 |
| Gras, J.,2020 | France | Western Europe | High |  | 901 | Antibody | Cohort | Convenient | 6 |
| Herbert, S.,2021 | Australia | Australia/New Zealand | High | 39.3 | 9457 | Antibody | Cohort | Convenient | 4 |
| Ho, S. Y.,2020 | Taiwan | Eastern Asia | High |  | 16362 | Antibody | Cohort | Convenient | 7 |
| Hoornenborg, E.,2020 | Netherlands | Western Europe | High | 40 | 654 | Antibody | Cohort | Convenient | 9 |
| Schmidbauer, C.,2020 | Austria | Western Europe | High |  | 4892 | Antibody or RNA | Cohort | Convenient | 9 |
| Tabatabavakili, S.,2021 | Canada | Northern America | High | 35 | 282 | Antibody | Cohort | Convenient | 8 |
| Vuylsteke, B.,2019 | Belgium | Western Europe | High | 38 | 318 | Antibody | Cohort | Convenient | 7 |
| ^#^Data of age are presented as median or mean. Adapted Newcastle Ottawa Scale (NOS) is used in quality assessment for Cohort, studies with scores of 7-9 are considered high quality, 4-6 are medium quality, 0-3 are low quality. | | | | | | | | | |

**Appendix 3 Forest plot of influential analysis by excluding each individual study on pooled estimates**

**
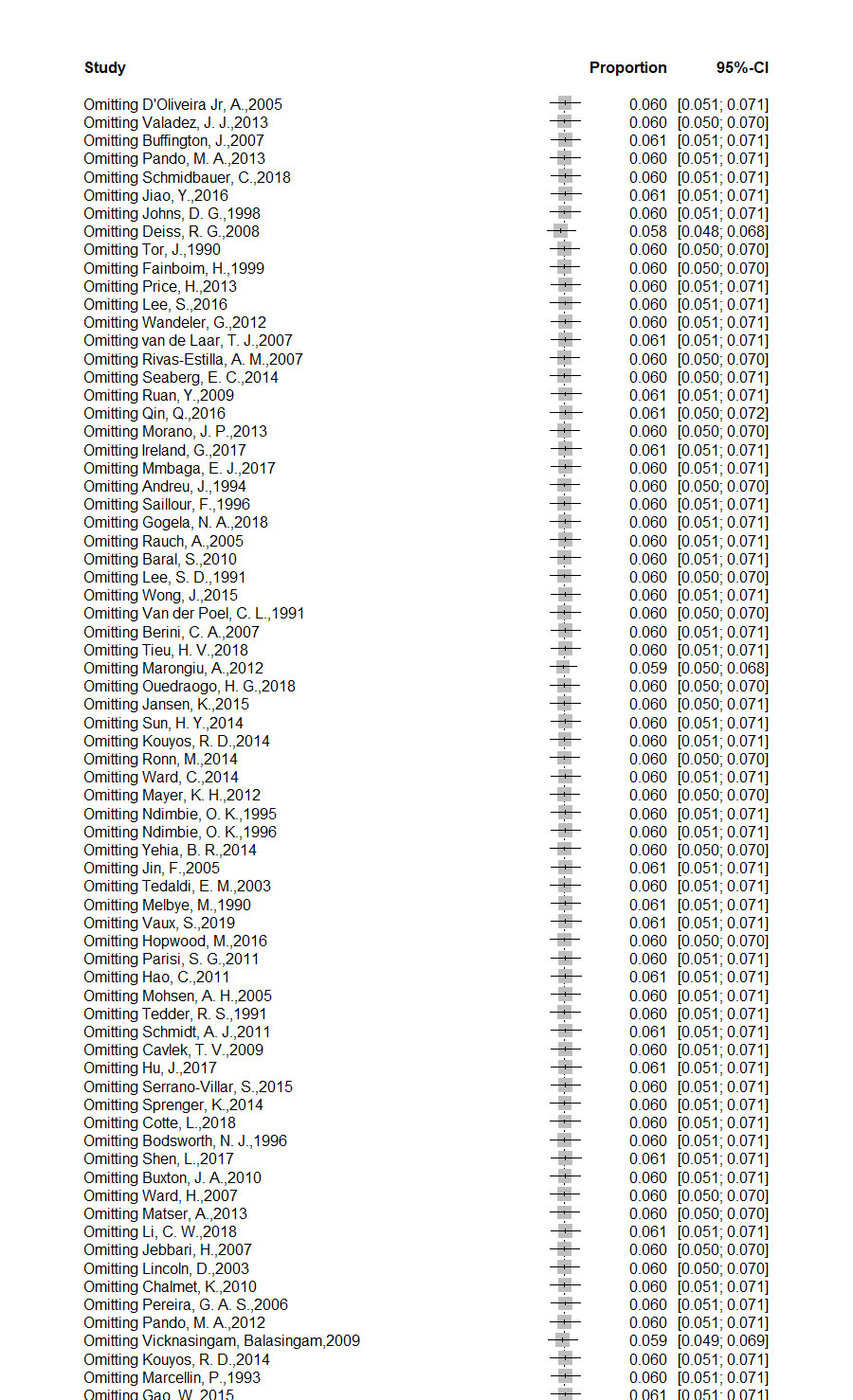

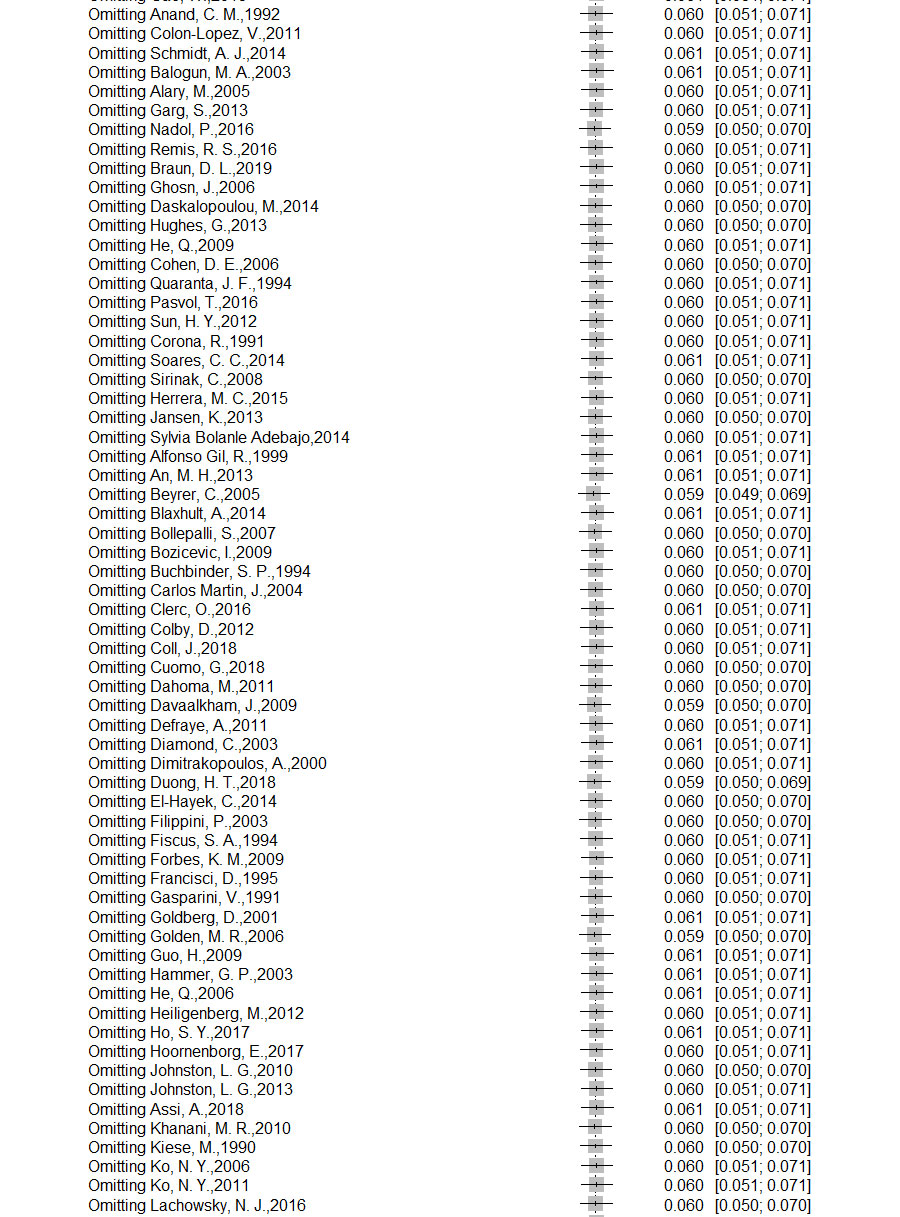

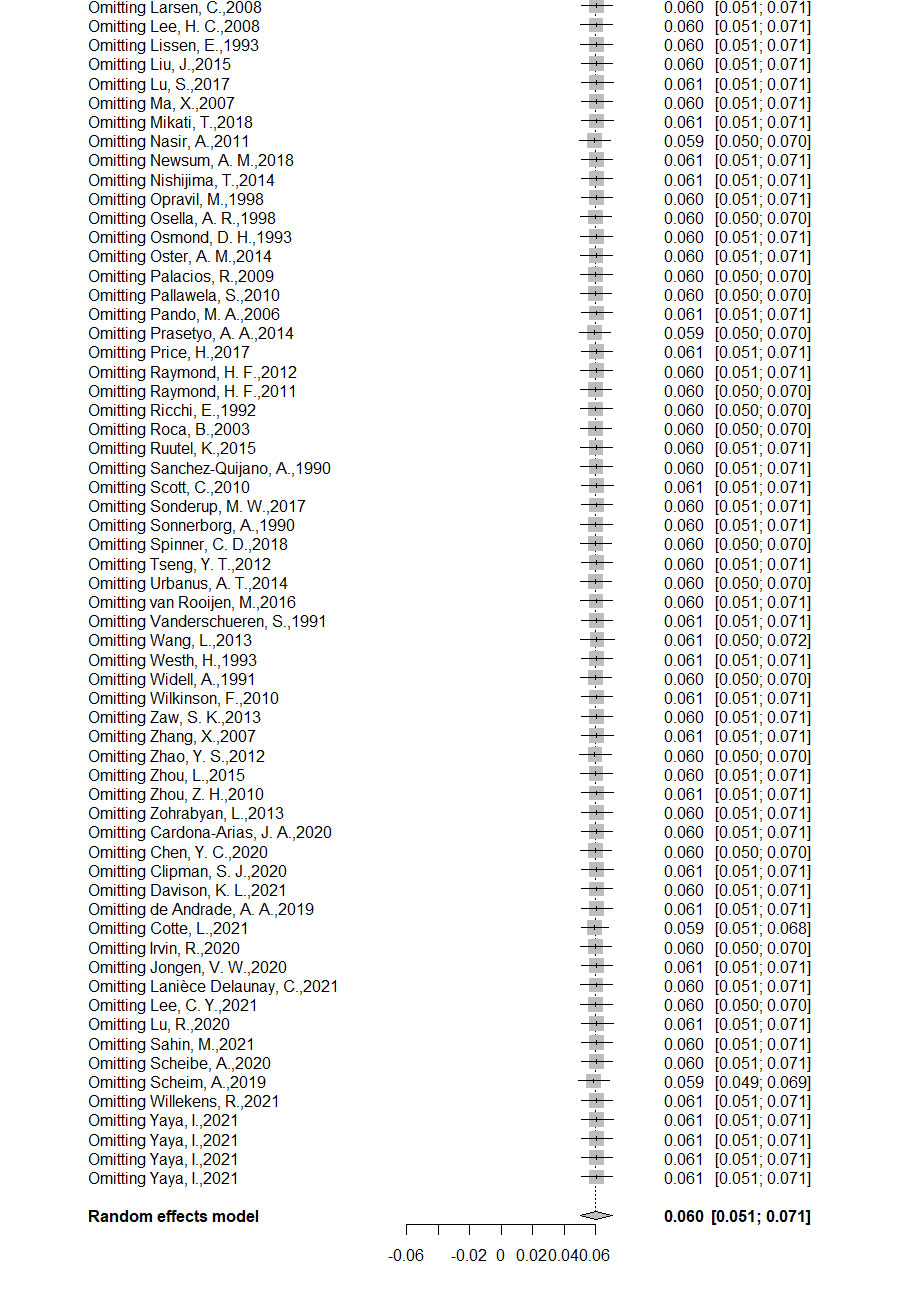
**

**Supplementary Figure 1. Forest plot of influential analysis on prevalence studies**

**
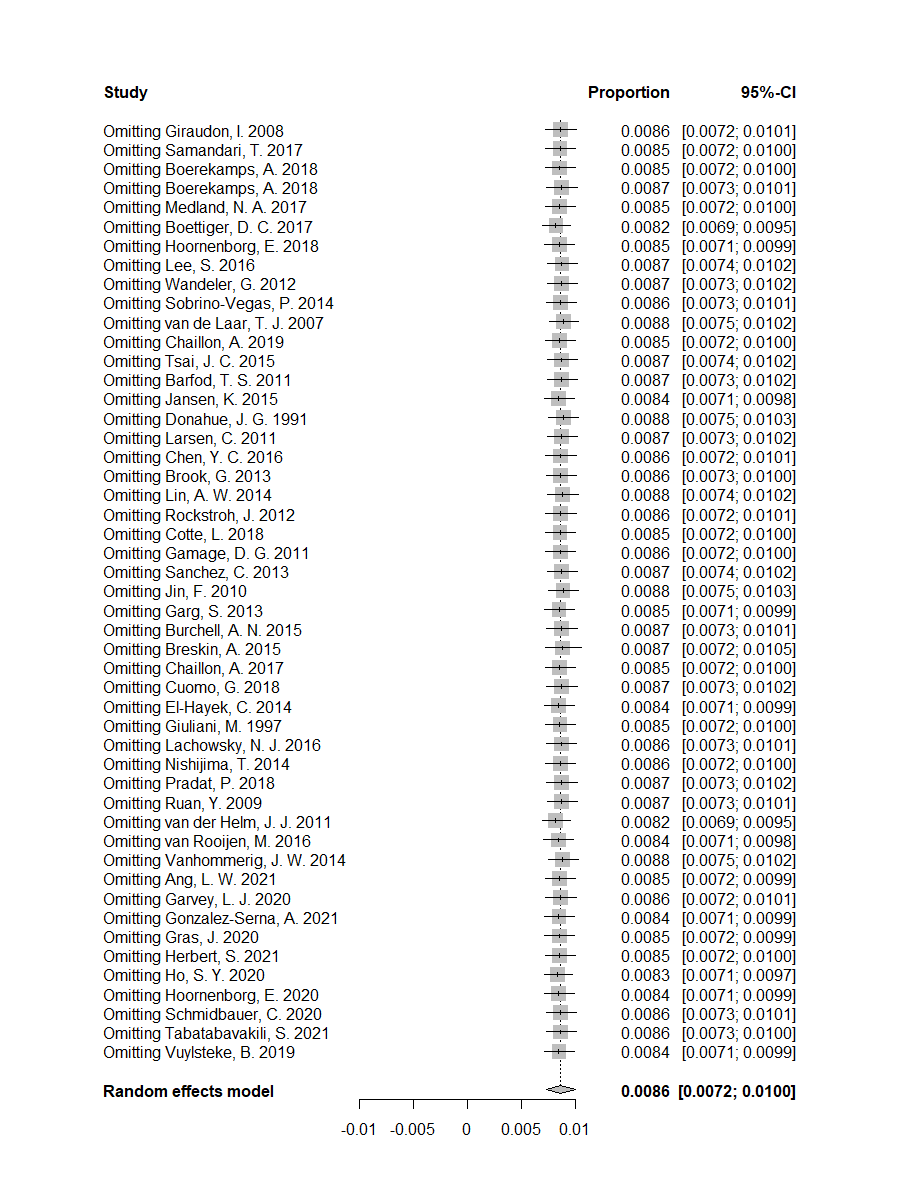
**

**Supplementary Figure 2. Forest plot of influential analysis on incidence studies**

**Appendix 4. Publication bias and funnel plot**

For prevalence study (included records, n=196)

Egger test for bias, p<0.05


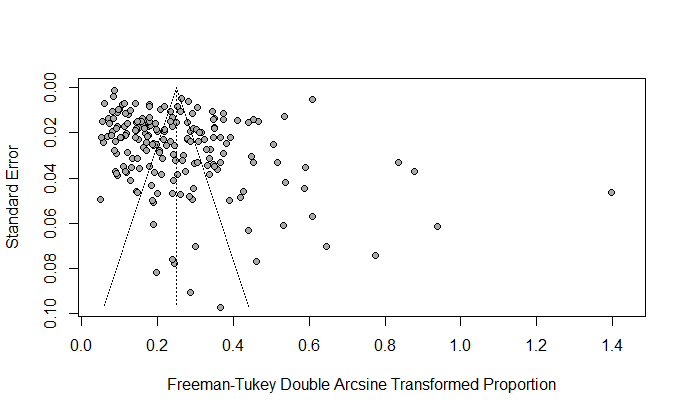


**Supplementary Figure 3-1. Funnel plot for prevalence studies**

Adjusted using trim-and-fill method

**
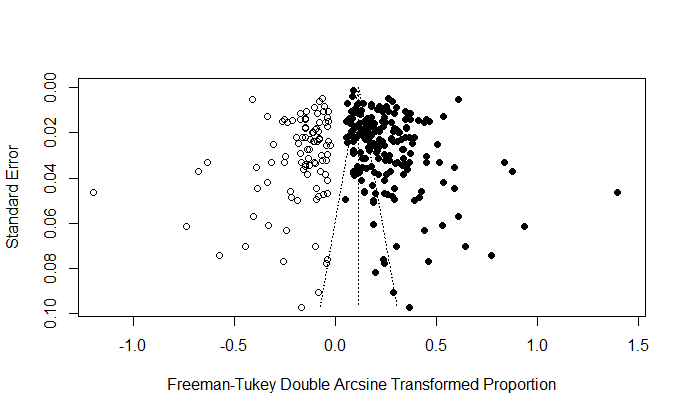
**

**Supplementary Figure 3-2. Funnel plot for prevalence studies**

For incidence study (included records, n=49)

Egger test for bias, p=0.1002


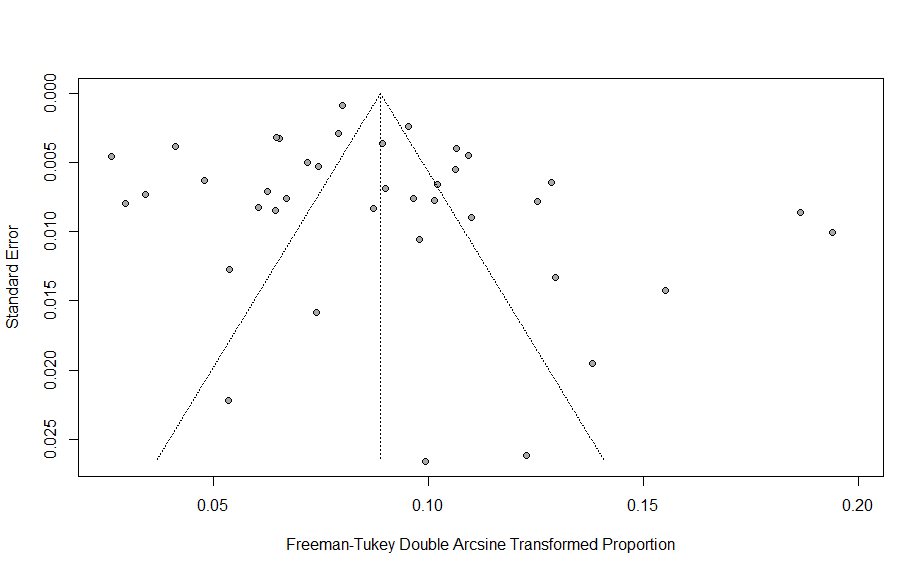


**Supplementary Figure 4. Funnel plot for incidence studies**

**Appendix 5. Forest plot of overall and subgroup prevalence/incidence**


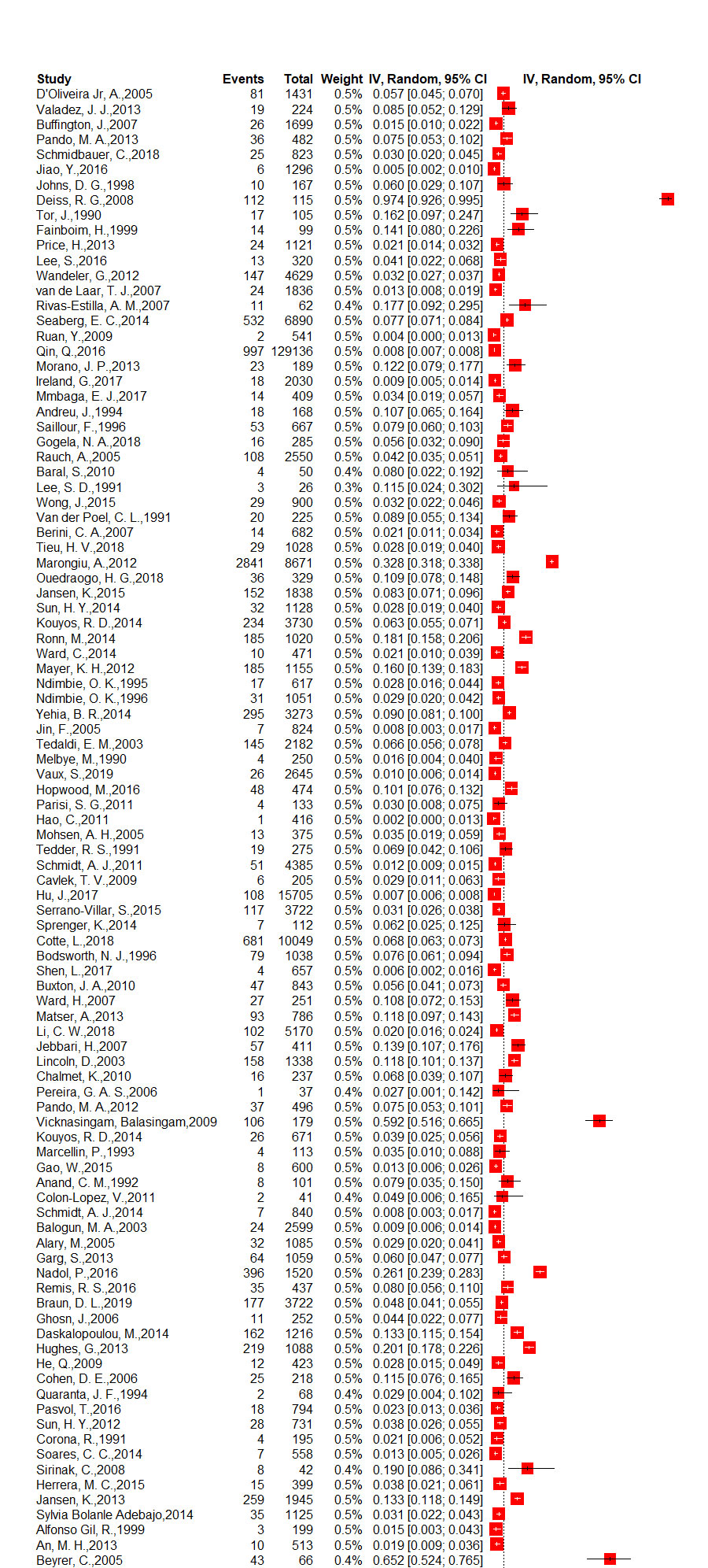


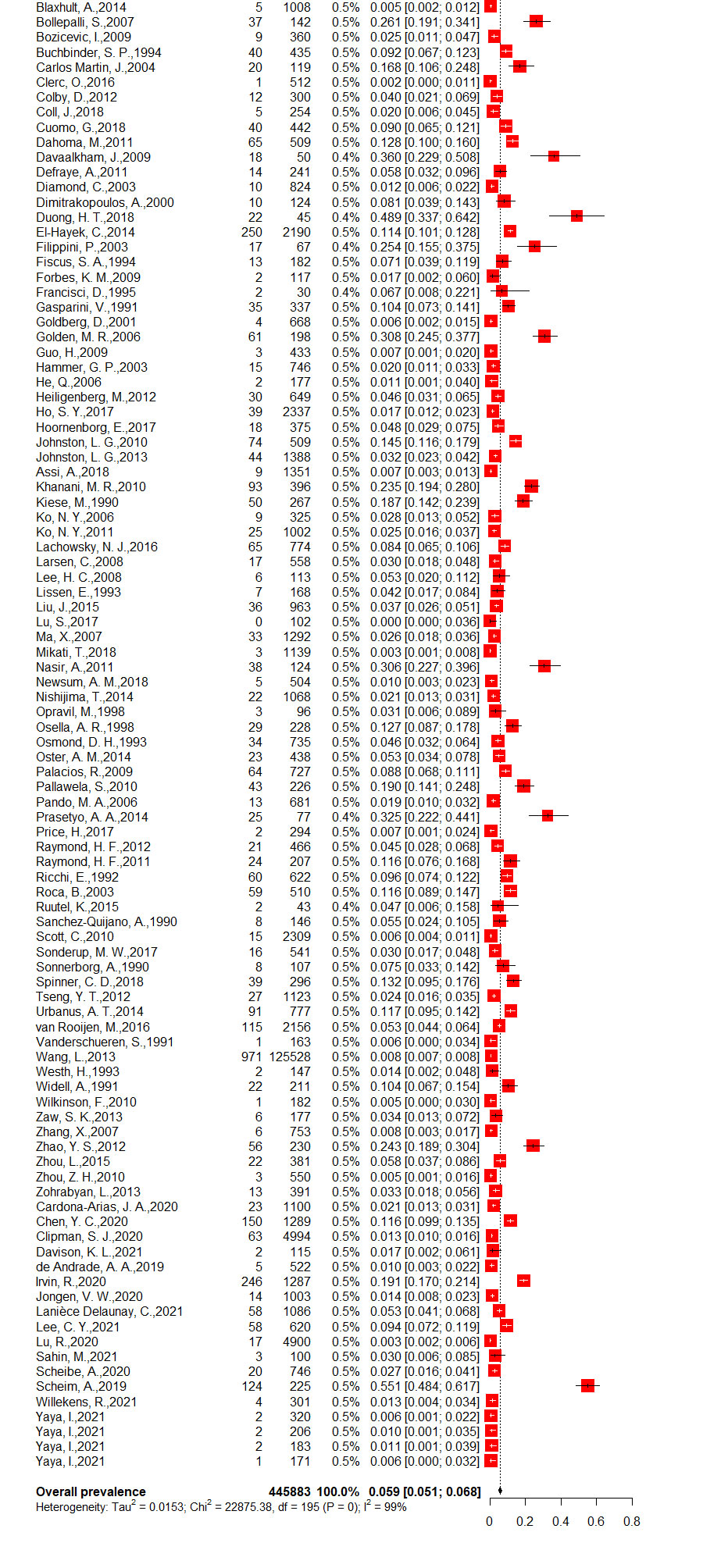


**Supplementary Figure 5. Forest plot of overall prevalence**

*HIV+ MSM*


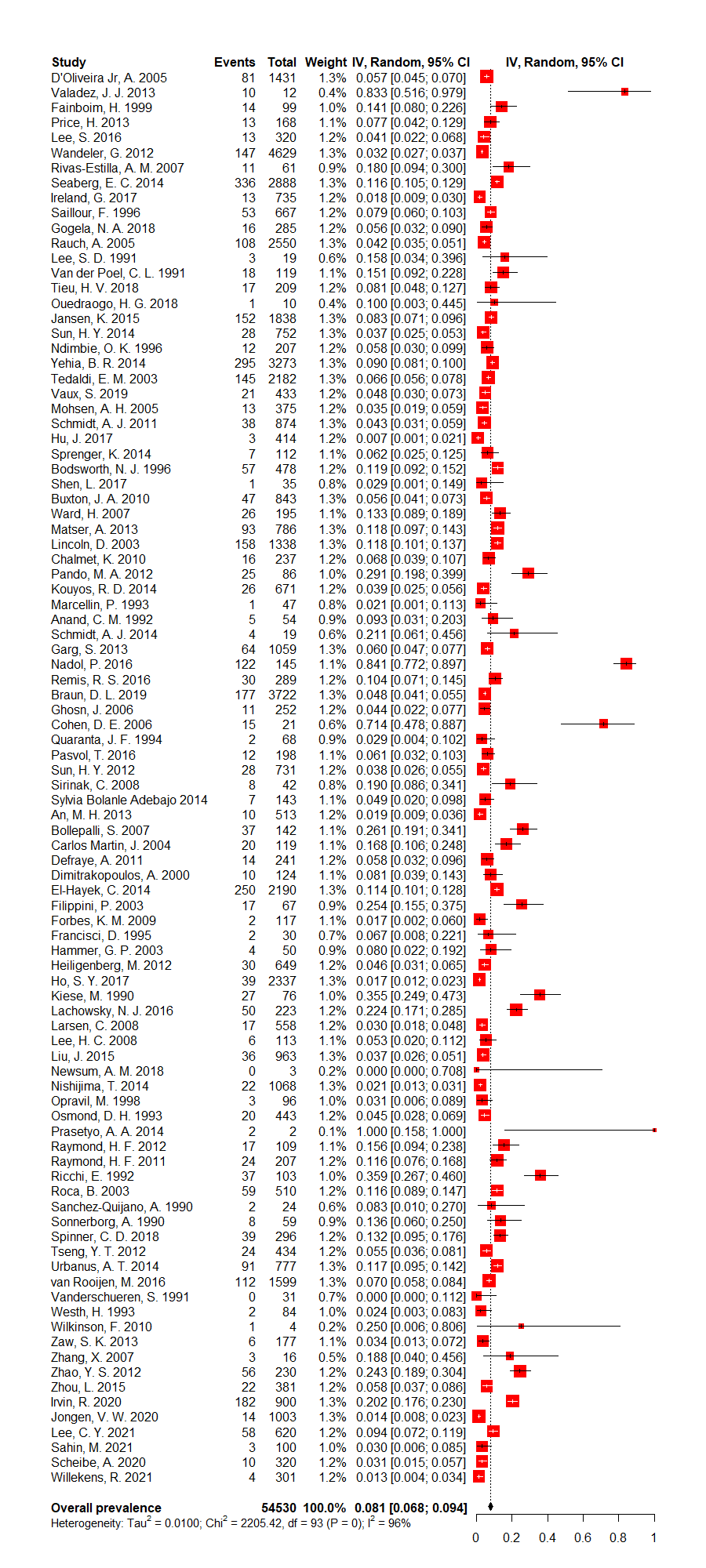


*HIV- MSM*


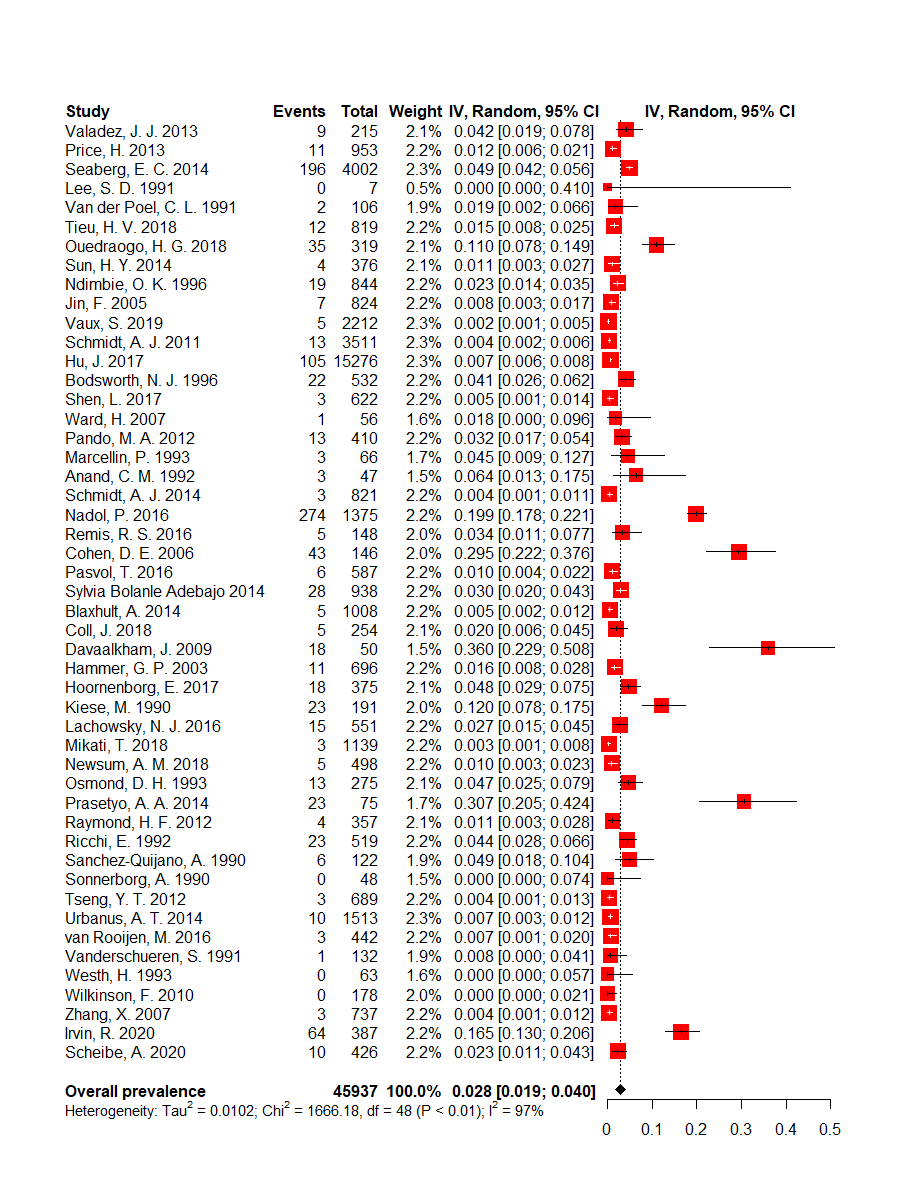


**Supplementary Figure 6. Forest plot of subgroup prevalence by HIV status**


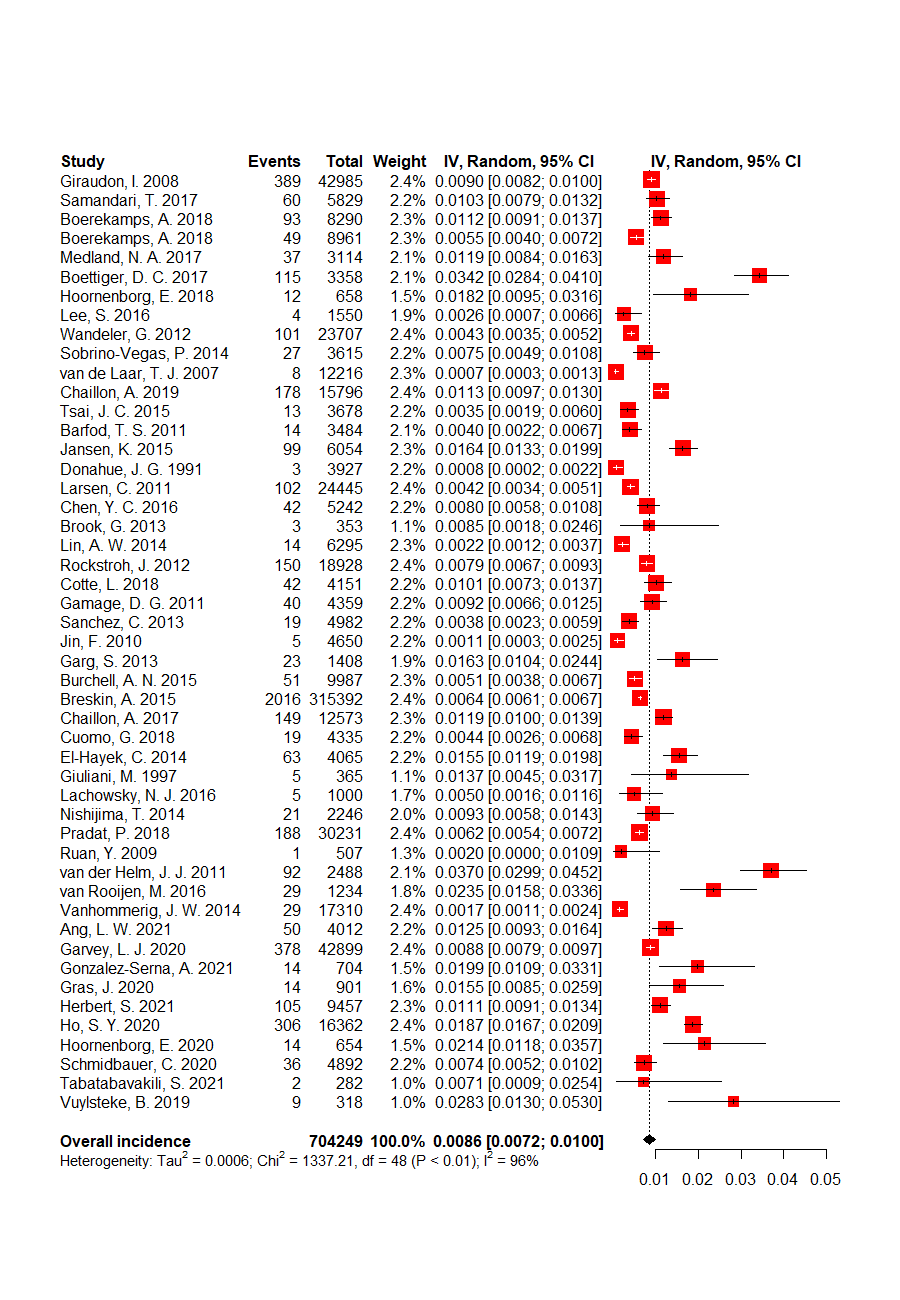


**Supplementary Figure 7. Forest plot of overall incidence**

**Appendix 6. List of included studies**

**Reference list 1: prevalence studies**

1. D'Oliveira Jr A, Voirin N, Allard R, et al. Prevalence and sexual risk of hepatitis C virus infection when human immunodeficiency virus was acquired through sexual intercourse among patients of the Lyon University Hospitals, France, 1992-2002. Journal of viral hepatitis 2005; 12:330-2.

2. Valadez JJ, Berendes S, Jeffery C, et al. Filling the Knowledge Gap: Measuring HIV Prevalence and Risk Factors among Men Who Have Sex with Men and Female Sex Workers in Tripoli, Libya. PloS one 2013; 8:e66701.

3. Buffington J, Murray PJ, Schlanger K, et al. Low prevalence of hepatitis C virus antibody in men who have sex with men who do not inject drugs. Public health reports (Washington, DC : 1974) 2007; 122 Suppl 2:63-7.

4. Pando MA, Balan IC, Dolezal C, et al. Low frequency of male circumcision and unwillingness to be circumcised among MSM in Buenos Aires, Argentina: association with sexually transmitted infections. Journal of the International AIDS Society 2013; 16:18500.

5. Schmidbauer C, Chromy D, Schmidbauer V, et al. Changing epidemiology of HCV coinfection among HIV+ patients. Hepatology (Baltimore, Md) 2018; 68:937A.

6. Jiao Y, Zhang X, Wang C, et al. Hepatitis C Virus Subtype and Evolution Characteristic Among Drug Users, Men Who Have Sex With Men, and the General Population in Beijing, China. Medicine 2016; 95:e2688.

7. Johns DG, Gill MJ. Seroprevalence of cytomegalovirus, Toxoplasma gondii, syphilis, and hepatitis B and C virus infections in a regional population seropositive for HIV infection. The Canadian journal of infectious diseases = Journal canadien des maladies infectieuses 1998; 9:209-14.

8. Deiss RG, Brouwer KC, Loza O, et al. High-risk sexual and drug using behaviors among male injection drug users who have sex with men in 2 Mexico-US border cities. Sexually transmitted diseases 2008; 35:243-9.

9. Tor J, Llibre JM, Carbonell M, et al. Sexual transmission of hepatitis C virus and its relation with hepatitis B virus and HIV. BMJ (Clinical research ed) 1990; 301:1130-3.

10. Fainboim H, Gonzalez J, Fassio E, et al. Prevalence of hepatitis viruses in an anti-human immunodeficiency virus-positive population from Argentina. A multicentre study. Journal of viral hepatitis 1999; 6:53-7.

11. Price H, Gilson R, Mercey D, et al. Hepatitis C in men who have sex with men in London--a community survey. HIV medicine 2013; 14:578-80.

12. Lee S, Lee SH, Lee SJ, et al. Incidence and risk factors of hepatitis C virus infection among human immunodeficiency virus (HIV) patients in a large HIV clinic in South Korea. The Korean journal of internal medicine 2016; 31:772-8.

13. Wandeler G, Gsponer T, Bregenzer A, et al. Hepatitis C virus infections in the Swiss HIV Cohort Study: a rapidly evolving epidemic. Clinical infectious diseases : an official publication of the Infectious Diseases Society of America 2012; 55:1408-16.

14. van de Laar TJ, van der Bij AK, Prins M, et al. Increase in HCV incidence among men who have sex with men in Amsterdam most likely caused by sexual transmission. The Journal of infectious diseases 2007; 196:230-8.

15. Rivas-Estilla AM, Ramirez-Valles E, Martinez-Hernandez R, et al. Hepatitis C virus infection among HIV-1 infected individuals from northern Mexico. Hepatology research : the official journal of the Japan Society of Hepatology 2007; 37:311-6.

16. Seaberg EC, Witt MD, Jacobson LP, et al. Differences in hepatitis C virus prevalence and clearance by mode of acquisition among men who have sex with men. Journal of viral hepatitis 2014; 21:696-705.

17. Ruan Y, Luo F, Jia Y, et al. Risk factors for syphilis and prevalence of HIV, hepatitis B and C among men who have sex with men in Beijing, China: implications for HIV prevention. AIDS and behavior 2009; 13:663-70.

18. Qin Q, Tang W, Ge L, et al. Changing trend of HIV, Syphilis and Hepatitis C among Men Who Have Sex with Men in China. Scientific reports 2016; 6:31081.

19. Morano JP, Gibson BA, Altice FL. The burgeoning HIV/HCV syndemic in the urban Northeast: HCV, HIV, and HIV/HCV coinfection in an urban setting. PloS one 2013; 8:e64321.

20. Ireland G, Higgins S, Goorney B, et al. Evaluation of hepatitis C testing in men who have sex with men, and associated risk behaviours, in Manchester, UK. Sexually transmitted infections 2017; 93:404-9.

21. Mmbaga EJ, Moen K, Makyao N, Mpembeni R, Leshabari MT. HIV and STI s among men who have sex with men in Dodoma municipality, Tanzania: a cross-sectional study. Sexually transmitted infections 2017; 93:314-9.

22. Andreu J, Abad MA, Sanchez-Quijano A, et al. High rate of nonspecific anti-hepatitis C reactivity amongst homosexual men in comparison with that found in other. Journal of internal medicine 1994; 236:73-7.

23. Saillour F, Dabis F, Dupon M, et al. Prevalence and determinants of antibodies to hepatitis C virus and markers for hepatitis B virus infection in patients with HIV infection in Aquitaine. Groupe d'Epidemiologie Clinique du SIDA en Aquitaine. BMJ (Clinical research ed) 1996; 313:461-4.

24. Gogela NA, Sonderup MW, Rebe K, Chivese T, Spearman CW. Hepatitis C prevalence in HIV-infected heterosexual men and men who have sex with men. South African medical journal = Suid-Afrikaanse tydskrif vir geneeskunde 2018; 108:568-72.

25. Rauch A, Rickenbach M, Weber R, et al. Unsafe sex and increased incidence of hepatitis C virus infection among HIV-infected men who have sex with men: the Swiss HIV Cohort Study. Clinical infectious diseases : an official publication of the Infectious Diseases Society of America 2005; 41:395-402.

26. Baral S, Kizub D, Masenior NF, et al. Male sex workers in Moscow, Russia: a pilot study of demographics, substance use patterns, and prevalence of HIV-1 and sexually transmitted infections. AIDS care 2010; 22:112-8.

27. Lee SD, Chan CY, Wang YJ, et al. Seroepidemiology of hepatitis C virus infection in Taiwan. Hepatology (Baltimore, Md) 1991; 13:830-3.

28. Wong J, Moore D, Kanters S, et al. Seroprevalence of hepatitis C and correlates of seropositivity among men who have sex with men in Vancouver, Canada: a cross-sectional survey. Sexually transmitted infections 2015; 91:430-3.

29. Van der Poel CL, Reesink HW, Mauser-Bunschoten EP, et al. Prevalence of anti-HCV antibodies confirmed by recombinant immunoblot in different population subsets in The Netherlands. Vox sanguinis 1991; 61:30-6.

30. Berini CA, Pando MA, Bautista CT, et al. HTLV-1/2 among high-risk groups in Argentina: molecular diagnosis and prevalence of different sexual transmitted infections. Journal of medical virology 2007; 79:1914-20.

31. Tieu HV, Laeyendecker O, Nandi V, et al. Prevalence and mapping of hepatitis C infections among men who have sex with men in New York City. PloS one 2018; 13:e0200269.

32. Marongiu A, Hope VD, Parry JV, Ncube F. Male IDUs who have sex with men in England, Wales and Northern Ireland: are they at greater risk of bloodborne virus infection and harm than those who only have sex with women? Sexually transmitted infections 2012; 88:456-61.

33. Ouedraogo HG, Kouanda S, Grosso A, et al. Hepatitis B, C, and D virus and human T-cell leukemia virus types 1 and 2 infections and correlates among men who have sex with men in Ouagadougou, Burkina Faso. Virology journal 2018; 15:194.

34. Jansen K, Thamm M, Bock CT, et al. High Prevalence and High Incidence of Coinfection with Hepatitis B, Hepatitis C, and Syphilis and Low Rate of Effective Vaccination against Hepatitis B in HIV-Positive Men Who Have Sex with Men with Known Date of HIV Seroconversion in Germany. PloS one 2015; 10:e0142515.

35. Sun HY, Cheng CY, Lee NY, et al. Seroprevalence of hepatitis B virus among adults at high risk for HIV transmission two decades after implementation of nationwide hepatitis B virus vaccination program in Taiwan. PloS one 2014; 9:e90194.

36. Kouyos RD, Rauch A, Boni J, et al. Clustering of HCV coinfections on HIV phylogeny indicates domestic and sexual transmission of HCV. International journal of epidemiology 2014; 43:887-96.

37. Ronn M, Hughes G, White P, Simms I, Ison C, Ward H. Characteristics of LGV repeaters: analysis of LGV surveillance data. Sexually transmitted infections 2014; 90:275-8.

38. Ward C, Lee V. Should we offer routine hepatitis C antibody testing in men who have sex with men? Journal of the International AIDS Society 2014; 17:19591.

39. Mayer KH, Ducharme R, Zaller ND, et al. Unprotected sex, underestimated risk, undiagnosed HIV and sexually transmitted diseases among men who have sex with men accessing testing services in a New England bathhouse. Journal of acquired immune deficiency syndromes (1999) 2012; 59:194-8.

40. Ndimbie OK, Nedjar S, Kingsley L, Riddle P, Rinaldo C. Long-term serologic follow-up of hepatitis C virus-seropositive homosexual men. Clinical and diagnostic laboratory immunology 1995; 2:219-24.

41. Ndimbie OK, Kingsley LA, Nedjar S, Rinaldo CR. Hepatitis C virus infection in a male homosexual cohort: risk factor analysis. Genitourinary medicine 1996; 72:213-6.

42. Yehia BR, Herati RS, Fleishman JA, et al. Hepatitis C virus testing in adults living with HIV: a need for improved screening efforts. PloS one 2014; 9:e102766.

43. Jin F, Prestage GP, Kippax SC, Kaldor JM, Dore GJ, Grulich AE. Prevalence and risk factors of hepatitis C in HIV-negative homosexual men in Sydney, Australia. Australian and New Zealand journal of public health 2005; 29:536-9.

44. Tedaldi EM, Huppler Hullsiek K, Malvestutto CD, et al. Prevalence and characteristics of hepatitis C virus coinfection in a human immunodefidency virus clinical trials group: The Terry Beirn Community Programs for Clinical Research on AIDS. Clinical Infectious Diseases 2003; 36:1313-7.

45. Melbye M, Biggar RJ, Wantzin P, Krogsgaard K, Ebbesen P, Becker NG. Sexual transmission of hepatitis C virus: cohort study (1981-9) among European homosexual men. BMJ (Clinical research ed) 1990; 301:210-2.

46. Vaux S, Chevaliez S, Saboni L, et al. Prevalence of hepatitis C infection, screening and associated factors among men who have sex with men attending gay venues: a cross-sectional survey (PREVAGAY), France, 2015. BMC infectious diseases 2019; 19:315.

47. Hopwood M, Lea T, Aggleton P. Multiple strategies are required to address the information and support needs of gay and bisexual men with hepatitis C in Australia. Journal of public health (Oxford, England) 2016; 38:156-62.

48. Parisi SG, Cruciani M, Scaggiante R, et al. Anal and oral human papillomavirus (HPV) infection in HIV-infected subjects in northern Italy: a longitudinal cohort study among men who have sex with men. BMC infectious diseases 2011; 11:150.

49. Hao C, Yan H, Yang H, et al. The incidence of syphilis, HIV and HCV and associated factors in a cohort of men who have sex with men in Nanjing, China. Sexually transmitted infections 2011; 87:199-201.

50. Mohsen AH, Murad S, Easterbrook PJ. Prevalence of hepatitis C in an ethnically diverse HIV-1-infected cohort in south London. HIV medicine 2005; 6:206-15.

51. Tedder RS, Gilson RJ, Briggs M, et al. Hepatitis C virus: evidence for sexual transmission. BMJ (Clinical research ed) 1991; 302:1299-302.

52. Schmidt AJ, Marcus U. Self-reported history of sexually transmissible infections (STIs) and STI-related utilization of the German health care system by men who have sex with men: data from a large convenience sample. BMC infectious diseases 2011; 11:132.

53. Cavlek TV, Margan IG, Lepej SZ, Kolaric B, Vince A. Seroprevalence, risk factors, and hepatitis C virus genotypes in groups with high-risk sexual behavior in Croatia. Journal of medical virology 2009; 81:1348-53.

54. Hu J, Gu X, Tao X, et al. Prevalence and Trends of HIV, Syphilis, and HCV in Migrant and Resident Men Who Have Sex with Men in Shandong, China: Results from a Serial Cross-Sectional Study. PloS one 2017; 12:e0170443.

55. Serrano-Villar S, Sobrino-Vegas P, Monge S, et al. Decreasing prevalence of HCV coinfection in all risk groups for HIV infection between 2004 and 2011 in Spain. Journal of viral hepatitis 2015; 22:496-503.

56. Sprenger K, Evison JM, Zwahlen M, et al. Sexually transmitted infections in HIV-infected people in Switzerland: cross-sectional study. PeerJ 2014; 2:e537.

57. Cotte L, Cua E, Reynes J, et al. Hepatitis C virus incidence in HIV-infected and in preexposure prophylaxis (PrEP)-using men having sex with men. Liver international : official journal of the International Association for the Study of the Liver 2018.

58. Bodsworth NJ, Cunningham P, Kaldor J, Donovan B. Hepatitis C virus infection in a large cohort of homosexually active men: independent associations with HIV-1 infection and injecting drug use but not sexual behaviour. Genitourinary medicine 1996; 72:118-22.

59. Shen L, Liu X, Fu G, et al. The Epidemic of Human Immunodeficiency Virus, Hepatitis C Virus, and Syphilis Infection, and the Correlates of Sexually Transmitted Infections among Men Who Have Sex with Men in Zhenjiang, Jiangsu, China. Japanese journal of infectious diseases 2017; 70:171-6.

60. Buxton JA, Yu A, Kim PH, et al. HCV co-infection in HIV positive population in British Columbia, Canada. BMC public health 2010; 10:225.

61. Ward H, Martin I, Macdonald N, et al. Lymphogranuloma venereum in the United kingdom. Clinical infectious diseases : an official publication of the Infectious Diseases Society of America 2007; 44:26-32.

62. Matser A, Vanhommerig J, Schim van der Loeff MF, et al. HIV-infected men who have sex with men who identify themselves as belonging to subcultures are at increased risk for hepatitis C infection. PloS one 2013; 8:e57740.

63. Li CW, Yang CJ, Sun HY, et al. Changing seroprevalence of hepatitis C virus infection among HIV-positive patients in Taiwan. PloS one 2018; 13:e0194149.

64. Jebbari H, Alexander S, Ward H, et al. Update on lymphogranuloma venereum in the United Kingdom. Sexually transmitted infections 2007; 83:324-6.

65. Lincoln D, Petoumenos K, Dore GJ. HIV/HBV and HIV/HCV coinfection, and outcomes following highly active antiretroviral therapy. HIV medicine 2003; 4:241-9.

66. Chalmet K, Staelens D, Blot S, et al. Epidemiological study of phylogenetic transmission clusters in a local HIV-1 epidemic reveals distinct differences between subtype B and non-B infections. BMC infectious diseases 2010; 10:262.

67. Pereira GAS, Stefani MMA, Martelli CMT, et al. Human immunodeficiency virus type 1 and hepatitis C virus co-infection and viral subtypes at an HIV Testing Center in Brazil. Journal of medical virology 2006; 78:719-23.

68. Pando MA, Balan IC, Marone R, et al. HIV and other sexually transmitted infections among men who have sex with men recruited by RDS in Buenos Aires, Argentina: high HIV and HPV infection. PloS one 2012; 7:e39834.

69. Vicknasingam B, Narayanan S, Navaratnam V. Prevalence rates and risk factors for hepatitis C among drug users not in treatment in Malaysia. Drug & Alcohol Review 2009; 28:447-54.

70. Kouyos RD, Rauch A, Braun DL, et al. Higher risk of incident hepatitis C virus coinfection among men who have sex with men, in whom the HIV genetic bottleneck at transmission was wide. The Journal of infectious diseases 2014; 210:1555-61.

71. Marcellin P, Colin JF, Martinot-Peignoux M, et al. Hepatitis C virus infection in anti-HIV positive and negative French homosexual men with chronic hepatitis: comparison of second- and third-generation anti-HCV testing. Liver 1993; 13:319-22.

72. Gao W, Li Z, Li Y, Qiao X. Sexual Practices and the Prevalence of HIV and Syphilis among Men Who Have Sex with Men in Lanzhou, China. Japanese journal of infectious diseases 2015; 68:370-5.

73. Anand CM, Fonseca K, Walle RP, Powell S, Williams M. Antibody to hepatitis C virus in selected groups of a Canadian urban population. International journal of epidemiology 1992; 21:142-5.

74. Colon-Lopez V, Rodriguez-Diaz CE, Ortiz AP, Soto-Salgado M, Suarez E, Perez CM. HIV-related risk behaviors among a sample of men who have sex with men in Puerto Rico: an overview of substance use and sexual practices. Puerto Rico health sciences journal 2011; 30:65-8.

75. Schmidt AJ, Falcato L, Zahno B, et al. Prevalence of hepatitis C in a Swiss sample of men who have sex with men: whom to screen for HCV infection? BMC public health 2014; 14:3.

76. Balogun MA, Ramsay ME, Parry JV, et al. A national survey of genitourinary medicine clinic attenders provides little evidence of sexual transmission of hepatitis C virus infection. Sexually transmitted infections 2003; 79:301-6.

77. Alary M, Joly JR, Vincelette J, Lavoie R, Turmel B, Remis RS. Lack of evidence of sexual transmission of hepatitis C virus in a prospective cohort study of men who have sex with men. American journal of public health 2005; 95:502-5.

78. Garg S, Taylor LE, Grasso C, Mayer KH. Prevalent and incident hepatitis C virus infection among HIV-infected men who have sex with men engaged in primary care in a Boston community health center. Clinical infectious diseases : an official publication of the Infectious Diseases Society of America 2013; 56:1480-7.

79. Nadol P, O'Connor S, Duong H, et al. High hepatitis C virus (HCV) prevalence among men who have sex with men (MSM) in Vietnam and associated risk factors: 2010 Vietnam Integrated Behavioural and Biologic Cross-Sectional Survey. Sexually transmitted infections 2016; 92:542-9.

80. Remis RS, Liu J, Loutfy MR, et al. Prevalence of Sexually Transmitted Viral and Bacterial Infections in HIV-Positive and HIV-Negative Men Who Have Sex with Men in Toronto. PloS one 2016; 11:e0158090.

81. Braun DL, Hampel B, Kouyos R, et al. High Cure Rates With Grazoprevir-Elbasvir With or Without Ribavirin Guided by Genotypic Resistance Testing Among Human Immunodeficiency Virus/Hepatitis C Virus-coinfected Men Who Have Sex With Men. Clinical infectious diseases : an official publication of the Infectious Diseases Society of America 2019; 68:569-76.

82. Ghosn J, Deveau C, Goujard C, et al. Increase in hepatitis C virus incidence in HIV-1-infected patients followed up since primary infection. Sexually transmitted infections 2006; 82:458-60.

83. Daskalopoulou M, Rodger A, Thornton A, et al. Sexual behaviour, recreational drug use and hepatitis C co-infection in HIV-diagnosed men who have sex with men in the United Kingdom: results from the ASTRA study. Journal of the International AIDS Society 2014; 17:19630.

84. Hughes G, Alexander S, Simms I, et al. Lymphogranuloma venereum diagnoses among men who have sex with men in the U.K.: interpreting a cross-sectional study using an epidemic phase-specific framework. Sexually transmitted infections 2013; 89:542-7.

85. He Q, Wang Y, Lin P, et al. High prevalence of risk behaviour concurrent with links to other high-risk populations: a potentially explosive HIV epidemic among men who have sex with men in Guangzhou, China. Sexually transmitted infections 2009; 85:383-90.

86. Cohen DE, Russell CJ, Golub SA, Mayer KH. Prevalence of hepatitis C virus infection among men who have sex with men at a Boston community health center and its association with markers of high-risk behavior. AIDS patient care and STDs 2006; 20:557-64.

87. Quaranta JF, Delaney SR, Alleman S, Cassuto JP, Dellamonica P, Allain JP. Prevalence of antibody to hepatitis C virus (HCV) in HIV-1-infected patients (nice SEROCO cohort). Journal of medical virology 1994; 42:29-32.

88. Pasvol T, Khan P, Thiagarajan A, Dakshina S, Sarner L, Orkin C. Low proportion of men who have sex with men (MSM) tested for hepatitis c despite high prevalence in 2 genito-urinary medicine (GUM) clinics. Sexually transmitted infections 2016; 92:A11.

89. Sun HY, Chang SY, Yang ZY, et al. Recent hepatitis C virus infections in HIV-infected patients in Taiwan: incidence and risk factors. Journal of clinical microbiology 2012; 50:781-7.

90. Corona R, Prignano G, Mele A, et al. Heterosexual and homosexual transmission of hepatitis C virus: Relation with hepatitis B virus and human immunodeficiency virus type 1. Epidemiology and infection 1991; 107:667-72.

91. Soares CC, Georg I, Lampe E, et al. HIV-1, HBV, HCV, HTLV, HPV-16/18, and Treponema pallidum infections in a sample of Brazilian men who have sex with men. PloS one 2014; 9:e102676.

92. Sirinak C, Kittikraisak W, Pinjeesekikul D, et al. Viral hepatitis and HIV-associated tuberculosis: risk factors and TB treatment outcomes in Thailand. BMC public health 2008; 8:245.

93. Herrera MC, Konda KA, Leon SR, et al. Hepatitis C infections among high-risk men who have sex with men and transgender women in Lima, Peru. Sexually transmitted infections 2015; 91:A181.

94. Jansen K, Scheufele R, Bock C, et al. High prevalence of hepatitis B (HBV) coinfections, and low rate of effective HBV-vaccination in msm with known date of HIV-1 seroconversion in Germany. Sexually transmitted infections 2013; 89.

95. Adebajo SB. Prevalence and correlates of HIV, syphilis, hepatitis B, hepatitis C infections and sexual behaviours of men who have sex with men in two cities in Nigeria. Vol. 78: ProQuest Information & Learning, 2017.

96. Alfonso Gil R, Hurtado Navarro I, Espacio Casanovas A, Santos Rubio G, Tomas Dols S. [Risk behaviours and seroprevalence to HIV, HBV and HCV in patients of the AIDS information and prevention center in Valencia, Spain]. Gaceta sanitaria 1999; 13:16-21.

97. An MH, Han XX, Liu J, et al. [Study on the rates of infection and spontaneous clearance on HCV among HIV-infected men who have sex with men in China]. Zhonghua liu xing bing xue za zhi = Zhonghua liuxingbingxue zazhi 2013; 34:15-8.

98. Beyrer C, Sripaipan T, Tovanabutra S, et al. High HIV, hepatitis C and sexual risks among drug-using men who have sex with men in northern Thailand. AIDS (London, England) 2005; 19:1535-40.

99. Blaxhult A, Samuelson A, Ask R, Hokeberg I. Limited spread of hepatitis C among HIV-negative men who have sex with men in Stockholm, Sweden. International journal of STD & AIDS 2014; 25:493-5.

100. Bollepalli S, Mathieson K, Bay C, et al. Prevalence of risk factors for hepatitis C virus in HIV-infected and HIV/hepatitis C virus-coinfected patients. Sexually transmitted diseases 2007; 34:367-70.

101. Bozicevic I, Rode OD, Lepej SZ, et al. Prevalence of sexually transmitted infections among men who have sex with men in Zagreb, Croatia. AIDS and behavior 2009; 13:303-9.

102. Buchbinder SP, Katz MH, Hessol NA, Liu J, O'Malley PM, Alter MJ. Hepatitis C virus infection in sexually active homosexual men. The Journal of infection 1994; 29:263-9.

103. Carlos Martin J, Castilla J, Lopez M, Arranz R, Gonzalez-Lahoz J, Soriano V. Impact of chronic hepatitis C on HIV-1 disease progression. HIV clinical trials 2004; 5:125-31.

104. Clerc O, Darling K, Calmy A, Dubois-Arber F, Cavassini M. Hepatitis C Virus Awareness Among Men Who Have Sex With Men in Southwest Switzerland. Sexually transmitted diseases 2016; 43:44-8.

105. Colby D, Trang NNN, Lan HTX, et al. Prevalence of sexually transmitted diseases, HIV, and hepatitis among male sex workers in Ho Chi Minh City, Vietnam. International Journal of Infectious Diseases 2012; 16:e332.

106. Coll J, Videla S, Leon A, et al. Early detection of HIV infection and of asymptomatic sexually transmitted infections among men who have sex with men. Clinical microbiology and infection : the official publication of the European Society of Clinical Microbiology and Infectious Diseases 2018; 24:540-5.

107. Cuomo G, Digaetano M, Menozzi M, et al. Incidence of HCV infection amongst HIV positive men who had sex with men and prevalence data from patients followed at the Infectious Diseases Clinic of Modena, Italy. Digestive and liver disease : official journal of the Italian Society of Gastroenterology and the Italian Association for the Study of the Liver 2018; 50:1334-8.

108. Dahoma M, Johnston LG, Holman A, et al. HIV and Related Risk Behavior Among Men Who Have Sex with Men in Zanzibar, Tanzania: Results of a Behavioral Surveillance Survey. AIDS and behavior 2011:1-7.

109. Davaalkham J, Unenchimeg P, Baigalmaa C, et al. High-risk status of HIV-1 infection in the very low epidemic country, Mongolia, 2007. International journal of STD & AIDS 2009; 20:391-4.

110. Defraye A, Van Beckhoven D, Sasse A. Surveillance of sexually transmitted infections among persons living with HIV. International journal of public health 2011; 56:169-74.

111. Diamond C, Thiede H, Perdue T, et al. Viral hepatitis among young men who have sex with men: prevalence of infection, risk behaviors, and vaccination. Sexually transmitted diseases 2003; 30:425-32.

112. Dimitrakopoulos A, Takou A, Haida A, Molangeli S, Gialeraki A, Kordossis T. The prevalence of hepatitis B and C in HIV-positive Greek patients: Relationship to survival of deceased AIDS patients. Journal of Infection 2000; 40:127-31.

113. Duong HT, Jarlais DD, Khuat OHT, et al. Risk Behaviors for HIV and HCV Infection Among People Who Inject Drugs in Hai Phong, Viet Nam, 2014. AIDS and behavior 2018; 22:2161-71.

114. El-Hayek C, Doyle JS, Cuevas M, et al. New hepatitis c infection and re-infection among HIV co-infected men in Melbourne, Australia. Journal of hepatology 2014; 60:S314.

115. Filippini P, Coppola N, Scolastico C, et al. Hepatitis viruses and HIV infection in the Naples area. Infezioni in Medicina 2003; 11:139-45.

116. Fiscus SA, Kelly WF, Battigelli DA, et al. Hepatitis C virus seroprevalence in clients of sexually transmitted disease clinics in North Carolina. Sexually transmitted diseases 1994; 21:155-60.

117. Forbes KM, Davis P, Sarner L. Introduction of a nurse-led sexual health service for HIV-positive men. International journal of STD & AIDS 2009; 20:54-5.

118. Francisci D, Baldelli F, Papili R, Stagni G, Pauluzzi S. Prevalence of HBV, HDV and HCV hepatitis markers in HIV-positive patients. European journal of epidemiology 1995; 11:123-6.

119. Gasparini V, Chiaramonte M, Moschen ME, et al. Hepatitis C virus infection in homosexual men: a seroepidemiological study in gay clubs in north-east Italy. European journal of epidemiology 1991; 7:665-9.

120. Goldberg D, Cameron S, Sharp G, et al. Hepatitis C virus among genitourinary clinic attenders in Scotland: unlinked anonymous testing. International journal of STD & AIDS 2001; 12:17-21.

121. Golden MR, Gift TL, Brewer DD, et al. Peer referral for HIV case-finding among men who have sex with men. AIDS (London, England) 2006; 20:1961-8.

122. Guo H, Wei JF, Yang H, Huan X, Tsui SK, Zhang C. Rapidly increasing prevalence of HIV and syphilis and HIV-1 subtype characterization among men who have sex with men in Jiangsu, China. Sexually transmitted diseases 2009; 36:120-5.

123. Hammer GP, Kellogg TA, McFarland WC, et al. Low incidence and prevalence of hepatitis C virus infection among sexually active non-intravenous drug-using adults, San Francisco, 1997-2000. Sexually transmitted diseases 2003; 30:919-24.

124. He Q, Wang Y, Lin P, et al. Potential bridges for HIV infection to men who have sex with men in Guangzhou, China. AIDS and behavior 2006; 10:S17-23.

125. Heiligenberg M, Rijnders B, Schim van der Loeff MF, et al. High prevalence of sexually transmitted infections in HIV-infected men during routine outpatient visits in the Netherlands. Sexually transmitted diseases 2012; 39:8-15.

126. Ho SY, Lin PH, Su LH, Su YC, Hung CC, Chang SY. HCV incidence among HIV-positive MSM in Taiwan during an outbreak of acute HCV. International Congress of Chemotherapy and Infection Conference 2017.

127. Hoornenborg E, Achterbergh RCA, Schim van der Loeff MF, et al. MSM starting preexposure prophylaxis are at risk of hepatitis C virus infection. AIDS (London, England) 2017; 31:1603-10.

128. Johnston LG, Holman A, Dahoma M, et al. HIV risk and the overlap of injecting drug use and high-risk sexual behaviours among men who have sex with men in Zanzibar (Unguja), Tanzania. The International journal on drug policy 2010; 21:485-92.

129. Johnston LG, Vaillant TC, Dolores Y, Vales HM. HIV, hepatitis B/C and syphilis prevalence and risk behaviors among gay, transsexuals and men who have sex with men, Dominican Republic. International journal of STD & AIDS 2013; 24:313-21.

130. Assi A, Abu Zaki S, Kinge N, Abou Abbas D, Naous J, Ghanem A, Aaraj R, Maher R, Bakouny Z, Azzi G, Tomb R. Prevalence of HIV and other sexually transmitted infections and their association with sexual behavior and substance use among 1351 MSM in Lebanon. International AIDS Society Conference 2018.

131. Khanani MR, Somani M, Khan S, Naseeb S, Ali SH. Prevalence of single, double, and triple infections of HIV, HCV and HBV among the MSM community in Pakistan. The Journal of infection 2010; 61:507-9.

132. Kiese M, Lenz K, Guggenmoos-Holzmann I, Stark K, Bienzle U. Epidemiology of hepatitis C in homosexual men. Klinische Wochenschrift 1990; 68:1082.

133. Ko NY, Lee HC, Chang JL, et al. Prevalence of human immunodeficiency virus and sexually transmitted infections and risky sexual behaviors among men visiting gay bathhouses in taiwan. Sexually transmitted diseases 2006; 33:467-73.

134. Ko NY, Lee HC, Hung CC, et al. Trends of HIV and sexually transmitted infections, estimated HIV incidence, and risky sexual behaviors among gay bathhouse attendees in Taiwan: 2004-2008. AIDS and behavior 2011; 15:292-7.

135. Lachowsky NJ, Stephenson K, Cui Z, et al. Prevalence and factors of HCV infection among HIV-negative and HIV-positive MSM. Topics in antiviral medicine 2016; 24:217.

136. Larsen C, Pialoux G, Salmon D, et al. Prevalence of hepatitis C and hepatitis B infection in the HIV-infected population of France, 2004. Euro surveillance : bulletin Europeen sur les maladies transmissibles = European communicable disease bulletin 2008; 13.

137. Lee HC, Ko NY, Lee NY, Chang CM, Ko WC. Seroprevalence of viral hepatitis and sexually transmitted disease among adults with recently diagnosed HIV infection in Southern Taiwan, 2000-2005: upsurge in hepatitis C virus infections among injection drug users. Journal of the Formosan Medical Association = Taiwan yi zhi 2008; 107:404-11.

138. Lissen E, Alter HJ, Abad MA, et al. Hepatitis C virus infection among sexually promiscuous groups and the heterosexual partners of hepatitis C virus infected index cases. European journal of clinical microbiology & infectious diseases : official publication of the European Society of Clinical Microbiology 1993; 12:827-31.

139. Liu J, Fan P, Xue X, et al. Prevalence of hepatitis C virus antibody among newly reported HIV infection cases in Henan, 2012-2014. Zhonghua liu xing bing xue za zhi = Zhonghua liuxingbingxue zazhi 2015; 36:1269-73.

140. Lu S, Cui Y, Guo W, Li DM, Sun J. [Epidemiological survey of prevalence of HIV infection, syphilis and hepatitis C in female sex workers and other 6 risk populations in Tibet Autonomous Region]. Zhonghua liu xing bing xue za zhi = Zhonghua liuxingbingxue zazhi 2017; 38:921-5.

141. Ma X, Zhang Q, He X, et al. Trends in prevalence of HIV, syphilis, hepatitis C, hepatitis B, and sexual risk behavior among men who have sex with men. Results of 3 consecutive respondent-driven sampling surveys in Beijing, 2004 through 2006. Journal of acquired immune deficiency syndromes (1999) 2007; 45:581-7.

142. Mikati T, Jamison K, Borges CM, Daskalakis DC. Low prevalence of hepatitis C virus among NYC MSM initiating PrEP and PEP, 2016-2017. Conference on Retroviruses and Opportunistic Infections Conference 2018.

143. Nasir A, Todd CS, Stanekzai MR, et al. Prevalence of HIV, hepatitis B and hepatitis C and associated risk behaviours amongst injecting drug users in three Afghan cities. International Journal of Drug Policy 2011; 22:109-19.

144. Newsum AM, van Rooijen MS, Kroone M, et al. Stable Low Hepatitis C Virus Antibody Prevalence Among HIV-Negative Men Who Have Sex With Men Attending the Sexually Transmitted Infection Outpatient Clinic in Amsterdam, 2007 to 2017. Sexually transmitted diseases 2018; 45:813-7.

145. Nishijima T, Shimbo T, Komatsu H, Hamada Y, Gatanaga H, Oka S. Incidence and risk factors for incident Hepatitis C infection among men who have sex with men with HIV-1 infection in a large Urban HIV clinic in Tokyo. Journal of acquired immune deficiency syndromes (1999) 2014; 65:213-7.

146. Opravil M, Hunziker R, Luthy R, Grob PJ. [Chronic hepatitis B and C in HIV-infected patients]. Deutsche medizinische Wochenschrift (1946) 1998; 123:753-60.

147. Osella AR, Massa MA, Joekes S, et al. Hepatitis B and C virus sexual transmission among homosexual men. The American journal of gastroenterology 1998; 93:49-52.

148. Osmond DH, Charlebois E, Sheppard HW, et al. Comparison of risk factors for hepatitis C and hepatitis B virus infection in homosexual men. The Journal of infectious diseases 1993; 167:66-71.

149. Oster AM, Sternberg M, Nebenzahl S, et al. Prevalence of HIV, sexually transmitted infections, and viral hepatitis by Urbanicity, among men who have sex with men, injection drug users, and heterosexuals in the United States. Sexually transmitted diseases 2014; 41:272-9.

150. Palacios R, Mata R, Aguilar I, et al. High seroprevalence but low incidence of HCV infection in a cohort of patients with sexually transmitted HIV in Andalusia, Spain. Journal of the International Association of Physicians in AIDS Care (Chicago, Ill : 2002) 2009; 8:100-5.

151. Pallawela S, Elgalib A, Almeida M, et al. Three weeks of doxycycline is an effective treatment for rectal lymphogranuloma venereum. British HIV Association and the British Association for Sexual Health and HIV Conference 2010.

152. Pando MA, Bautista CT, Maulen S, et al. Epidemiology of human immunodeficiency virus, viral hepatitis (B and C), treponema pallidum, and human T-cell lymphotropic I/II virus among men who have sex with men in Buenos Aires, Argentina. Sexually transmitted diseases 2006; 33:307-13.

153. Prasetyo AA, Ariapramuda R, Kindi EA, et al. Men having sex with men in Surakarta, Indonesia: demographics, behavioral characteristics and prevalence of blood borne pathogens. The Southeast Asian journal of tropical medicine and public health 2014; 45:1032-47.

154. Price H, Salimee S, Coelho D. Prevalence of hepatitis B and hepatitis C in a UK genitourinary medicine clinic. International journal of STD & AIDS 2017; 28:238-41.

155. Raymond HF, Chu P, Nieves-Rivera I, Louie B, McFarland W, Pandori M. Hepatitis C infection among men who have sex with men, San Francisco, 2011. Sexually transmitted diseases 2012; 39:985-6.

156. Raymond HF, Hughes A, O'Keefe K, Stall RD, McFarland W. Hepatitis C prevalence among HIV-positive MSM in San Francisco: 2004 and 2008. Sexually transmitted diseases 2011; 38:219-20.

157. Ricchi E, Borderi M, Costigliola P, Miniero R, Sprovieri G, Chiodo F. Anti-hepatitis C virus antibodies amongst Italian homo-bisexual males. European journal of epidemiology 1992; 8:804-7.

158. Roca B, Suarez I, Gonzalez J, et al. Hepatitis C virus and human immunodeficiency virus coinfection in Spain. Journal of Infection 2003; 47:117-24.

159. Ruutel K, Lohmus L, Janes J. Internet-based recruitment system for HIV and STI screening for men who have sex with men in Estonia, 2013: analysis of preliminary outcomes. Euro surveillance : bulletin Europeen sur les maladies transmissibles = European communicable disease bulletin 2015; 20.

160. Sanchez-Quijano A, Rey C, Aguado I, et al. Hepatitis C virus infection in sexually promiscuous groups. European journal of clinical microbiology & infectious diseases : official publication of the European Society of Clinical Microbiology 1990; 9:610-2.

161. Scott C, Day S, Low E, Sullivan A, Atkins M, Asboe D. Unselected hepatitis C screening of men who have sex with men attending sexual health clinics. The Journal of infection 2010; 60:351-3.

162. Sonderup MW, Prabdial-Singh N, Manamela MJ, et al. Characteristics of hepatitis B and c prevalence in key populations in South Africa. American Association for the Study of Liver Diseases Conference 2017.

163. Sonnerborg A, Abebe A, Strannegard O. Hepatitis C virus infection in individuals with or without human immunodeficiency virus type 1 infection. Infection 1990; 18:347-51.

164. Spinner CD, Boesecke C, Jordan C, et al. Prevalence of asymptomatic sexually transmitted infections in HIV-positive men who have sex with men in Germany: results of a multicentre cross-sectional study. Infection 2018; 46:341-7.

165. Tseng YT, Sun HY, Chang SY, et al. Seroprevalence of hepatitis virus infection in men who have sex with men aged 18-40 years in Taiwan. Journal of the Formosan Medical Association = Taiwan yi zhi 2012; 111:431-8.

166. Urbanus AT, Van De Laar TJ, Geskus R, et al. Trends in hepatitis C virus infections among MSM attending a sexually transmitted infection clinic; 1995-2010. AIDS (London, England) 2014; 28:781-90.

167. van Rooijen M, Heijman T, de Vrieze N, et al. Earlier Detection of Hepatitis C Virus Infection Through Routine Hepatitis C Virus Antibody Screening of Human Immunodeficiency Virus-Positive Men Who Have Sex With Men Attending A Sexually Transmitted Infection Outpatient Clinic: A Longitudinal Study. Sexually transmitted diseases 2016; 43:560-5.

168. Vanderschueren S, Van Renterghem L, Plum J, Verhofstede C, Mak R, Vincke J. Hepatitis C among risk groups for HIV and hepatitis B. International journal of STD & AIDS 1991; 2:185-7.

169. Wang L, Li DM, Ge L, et al. [HCV prevalence among the populations under the HIV sentinel surveillance data from 2009 to 2012 in China]. Zhonghua liu xing bing xue za zhi = Zhonghua liuxingbingxue zazhi 2013; 34:543-7.

170. Westh H, Worm AM, Jensen BL, et al. Hepatitis C virus antibodies in homosexual men and intravenous drug users in Denmark. Infection 1993; 21:115-7.

171. Widell A, Hansson BG, Berntorp E, et al. Antibody to a hepatitis C virus related protein among patients at high risk for hepatitis B. Scandinavian journal of infectious diseases 1991; 23:19-24.

172. Wilkinson F, Riddell L, Noland D, Ghanem M. Should we be testing for hepatitis C routinely in men who have sex with men MSM in a non-urban sexual health clinic? British HIV Association and the British Association for Sexual Health and HIV Conference 2010.

173. Zaw SK, Tun ST, Thida A, et al. Prevalence of hepatitis C and B virus among patients infected with HIV: a cross-sectional analysis of a large HIV care programme in Myanmar. Tropical doctor 2013; 43:113-5.

174. Zhang X, Wang C, Hengwei W, et al. Risk factors of HIV infection and prevalence of co-infections among men who have sex with men in Beijing, China. AIDS (London, England) 2007; 21 Suppl 8:S53-7.

175. Zhao YS, Su SI, Lv CX, et al. Seroprevalence of hepatitis C, hepatitis B virus and syphilis in HIV-1 infected patients in Shandong, China. International journal of STD & AIDS 2012; 23:639-43.

176. Zhou L, Wu Q, Shen W, et al. Co-infection of hepatitis C virus among newly diagnosed HIV-infected adults in Taizhou prefecture of Zhejiang province, China. Chinese Journal of Endemiology 2015; 36:862-6.

177. Zhou ZH, Li SM, Liu YJ, et al. [Study on the relationship between behavioral factors, psychological status and HIV infection among men who have sex with men in Beijing.]. Zhonghua liu xing bing xue za zhi = Zhonghua liuxingbingxue zazhi 2010; 31:273-6.

178. Zohrabyan L, Johnston L, Scutelniciuc O, et al. HIV, hepatitis and syphilis prevalence and correlates of condom use during anal sex among men who have sex with men in the Republic of Moldova. International journal of STD & AIDS 2013; 24:357-64.

179. Cardona-Arias J A, Correa J C C, Higuita-Gutiérrez L F. Prevalence of hepatitis B/C viruses and associated factors in key groups attending a health services institution in Colombia, 2019. PloS one, 2020, 15(9): e0238655.

180. Chen Y C, Thio C L, Kamangar F, et al. Evolving trends in the prevalence of hepatitis C virus antibody positivity among HIV‐infected men in a community‐based primary care setting. Journal of viral hepatitis, 2020, 27(11): 1202-1213.

181. Clipman S J, Duggal P, Srikrishnan A K, et al. Prevalence and Phylogenetic Characterization of Hepatitis C Virus Among Indian Men Who Have Sex With Men: Limited Evidence for Sexual Transmission. The Journal of infectious diseases, 2020, 221(11): 1875-1883.

182. Davison K L, Reynolds C A, Andrews N, et al. Blood donation by men who have sex with men: using evidence to change policy. Vox Sanguinis, 2021, 116(3): 260-272.

183. Andrade A A, Carneiro M A S, Teles S A, et al. Hepatitis C prevalence among men who have sex with men in Central Brazil. Brazilian Journal of Infectious Diseases, 2019, 23: 271-273.

184. Irvin R, Gamble T, Malone J, et al. HPTN 078: high prevalence of HCV antibodies among urban US men who have sex with men (MSM) independent of HIV status. Clin Infect Dis, 2020.

185. Jongen V W, van Rooijen M S, van der Loeff M F S, et al. Evaluation of the Hepatitis C Testing Strategy for Human Immunodeficiency Virus–Positive Men Who Have Sex With Men at the Sexually Transmitted Infections Outpatient Clinic of Amsterdam, the Netherlands. Sexually transmitted diseases, 2020, 47(9): 587-595.

186. Delaunay C L, Cox J, Klein M, et al. Trends in hepatitis C virus seroprevalence and associated risk factors among men who have sex with men in Montréal: results from three cross-sectional studies (2005, 2009, 2018). Sexually transmitted infections, 2021, 97(4): 290-296.

187. Lee C Y, Wu P H, Lu M W, et al. High prevalence of unawareness of HCV infection status among both HCV-seronegative and seropositive people living with human immunodeficiency virus in Taiwan. PloS one, 2021, 16(5): e0251158.

188. Lu R, Zhang X, Zhou C, et al. Trends of human immunodeficiency virus, syphilis, and hepatitis C infections among men who have sex with men in Chongqing, China: A serial cross-sectional survey from 2011 to 2018. Sexually Transmitted Diseases, 2020, 47(7): 491.

189. Şahin M, Aydın Ö A, Karaosmanoğlu H K, et al. Seroprevalence of HBsAg and Anti-HCV among HIV Positive Patients. Viral Hepatit Dergisis, 2021, 27(1): 24.

190. Scheibe A, Young K, Versfeld A, et al. Hepatitis B, hepatitis C and HIV prevalence and related sexual and substance use risk practices among key populations who access HIV prevention, treatment and related services in South Africa: findings from a seven-city cross-sectional survey (2017). BMC Infectious Diseases, 2020, 20(1): 1-15.

191. Scheim A, Knight R, Shulha H, et al. Characterizing men who have sex with men and use injection drugs in Vancouver, Canada. AIDS and Behavior, 2019, 23(12): 3324-3330.

192. Willekens R, Sánchez I, Miguel L, et al. Screening for asymptomatic STIs in HIV-infected men who have sex with men. Sexually Transmitted Infections, 2021, 97(2): 170-171.

193. Yaya I, Dembélé Keita B, Anoma C, et al. Low Seroprevalence of Hepatitis C Among Men Who Have Sex With Men in West Africa. Clinical Infectious Diseases, 2021.

**Reference list 2: Incidence studies**

1. Giraudon I, Ruf M, Maguire H, et al. Increase in diagnosed newly acquired hepatitis C in HIV-positive men who have sex with men across London and Brighton, 2002-2006: is this an outbreak? Sexually transmitted infections 2008; 84:111-5.

2. Samandari T, Tedaldi E, Armon C, et al. Incidence of Hepatitis C Virus Infection in the Human Immunodeficiency Virus Outpatient Study Cohort, 2000-2013. Open forum infectious diseases 2017; 4:ofx076.

3. Boerekamps A, van den Berk GE, Lauw FN, et al. Declining Hepatitis C Virus (HCV) Incidence in Dutch Human Immunodeficiency Virus-Positive Men Who Have Sex With Men After Unrestricted Access to HCV Therapy. Clinical infectious diseases : an official publication of the Infectious Diseases Society of America 2018; 66:1360-5.

4. Medland NA, Chow EP, Bradshaw CS, Read TH, Sasadeusz JJ, Fairley CK. Predictors and incidence of sexually transmitted Hepatitis C virus infection in HIV positive men who have sex with men. BMC infectious diseases 2017; 17:185.

5. Boettiger DC, Law MG, Dore GJ, et al. Hepatitis C testing and re-testing among people attending sexual health services in Australia, and hepatitis C incidence among people with human immunodeficiency virus: analysis of national sentinel surveillance data. BMC infectious diseases 2017; 17:740.

6. Hoornenborg E, Coyer L, Achterbergh R, et al. High incidence of hepatitis C virus (re) infections among PrEP users in the Netherlands: Implications for prevention, monitoring and treatment. Journal of viral hepatitis 2018; 25:192.

7. Lee S, Lee SH, Lee SJ, et al. Incidence and risk factors of hepatitis C virus infection among human immunodeficiency virus (HIV) patients in a large HIV clinic in South Korea. The Korean journal of internal medicine 2016; 31:772-8.

8. Wandeler G, Gsponer T, Bregenzer A, et al. Hepatitis C virus infections in the Swiss HIV Cohort Study: a rapidly evolving epidemic. Clinical infectious diseases : an official publication of the Infectious Diseases Society of America 2012; 55:1408-16.

9. Sobrino-Vegas P, Monge Corella S, Serrano-Villar S, et al. Incidence of hepatitis C virus (HCV) in a multicenter cohort of HIV-positive patients in Spain 2004-2011: increasing rates of HCV diagnosis but not of HCV seroconversions. PloS one 2014; 9:e116226.

10. van de Laar TJ, van der Bij AK, Prins M, et al. Increase in HCV incidence among men who have sex with men in Amsterdam most likely caused by sexual transmission. The Journal of infectious diseases 2007; 196:230-8.

11. Chaillon A, Sun X, Cachay ER, et al. Primary Incidence of Hepatitis C Virus Infection Among HIV-Infected Men Who Have Sex With Men in San Diego, 2000-2015. Open forum infectious diseases 2019; 6:ofz160.

12. Tsai JC, Hung CC, Chang SY, et al. Increasing incidence of recent hepatitis C virus infection among persons seeking voluntary counselling and testing for HIV and sexually transmitted infections in Taiwan. BMJ open 2015; 5:e008406.

13. Barfod TS, Omland LH, Katzenstein TL. Incidence and characteristics of sexually transmitted acute hepatitis C virus infection among HIV-positive men who have sex with men in Copenhagen, Denmark during four years (2006-2009): a retrospective cohort study. Scandinavian journal of infectious diseases 2011; 43:145-8.

14. Jansen K, Thamm M, Bock CT, et al. High Prevalence and High Incidence of Coinfection with Hepatitis B, Hepatitis C, and Syphilis and Low Rate of Effective Vaccination against Hepatitis B in HIV-Positive Men Who Have Sex with Men with Known Date of HIV Seroconversion in Germany. PloS one 2015; 10:e0142515.

15. Donahue JG, Nelson KE, Munoz A, et al. Antibody to hepatitis C virus among cardiac surgery patients, homosexual men, and intravenous drug users in Baltimore, Maryland. American journal of epidemiology 1991; 134:1206-11.

16. Larsen C, Chaix ML, Le Strat Y, et al. Gaining greater insight into HCV emergence in HIV-infected men who have sex with men: the HEPAIG Study. PloS one 2011; 6:e29322.

17. Chen YC, Wiberg KJ, Hsieh YH, et al. Favorable Socioeconomic Status and Recreational Polydrug Use Are Linked With Sexual Hepatitis C Virus Transmission Among Human Immunodeficiency Virus-Infected Men Who Have Sex With Men. Open forum infectious diseases 2016; 3:ofw137.

18. Brook G, McSorley J, Shaw A. Retrospective study of the effect of enhanced systematic sexually transmitted infection screening, facilitated by the use of electronic patient records, in an HIV-infected cohort. HIV medicine 2013; 14:347-53.

19. Lin AW, Wong KH, Chan K. More safer sex intervention needed for HIV-positive MSM with higher education level for prevention of sexually transmitted hepatitis C. Journal of the International AIDS Society 2014; 17:19663.

20. Rockstroh J, Grint D, Boesecke C, et al. Increases in acute hepatitis C (HCV) incidence across Europe: Which regions and patient groups are affected? Journal of the International AIDS Society 2012; 15:10.

21. Cotte L, Cua E, Reynes J, et al. Hepatitis C virus incidence in HIV-infected and in preexposure prophylaxis (PrEP)-using men having sex with men. Liver international : official journal of the International Association for the Study of the Liver 2018.

22. Gamage DG, Read TR, Bradshaw CS, et al. Incidence of hepatitis-C among HIV infected men who have sex with men (MSM) attending a sexual health service: a cohort study. BMC infectious diseases 2011; 11:39.

23. Sanchez C, Plaza Z, Vispo E, et al. Scaling up epidemics of acute hepatitis C and syphilis in HIV-infected men who have sex with men in Spain. Liver international : official journal of the International Association for the Study of the Liver 2013; 33:1357-62.

24. Jin F, Prestage GP, Matthews G, et al. Prevalence, incidence and risk factors for hepatitis C in homosexual men: data from two cohorts of HIV-negative and HIV-positive men in Sydney, Australia. Sexually transmitted infections 2010; 86:25-8.

25. Garg S, Taylor LE, Grasso C, Mayer KH. Prevalent and incident hepatitis C virus infection among HIV-infected men who have sex with men engaged in primary care in a Boston community health center. Clinical infectious diseases : an official publication of the Infectious Diseases Society of America 2013; 56:1480-7.

26. Burchell AN, Gardner SL, Mazzulli T, et al. Hepatitis C virus seroconversion among HIV-positive men who have sex with men with no history of injection drug use: Results from a clinical HIV cohort. The Canadian journal of infectious diseases & medical microbiology = Journal canadien des maladies infectieuses et de la microbiologie medicale 2015; 26:17-22.

27. Breskin A, Drobnik A, Pathela P, et al. Factors Associated With Hepatitis C Infection Among HIV-Infected Men Who Have Sex With Men With No Reported Injection Drug Use in New York City, 2000-2010. Sexually transmitted diseases 2015; 42:382-6.

28. Chaillon A, Anderson CM, Martin TC, et al. Incidence of hepatitis C among HIV-infected men who have sex with men, 2000-2015. Topics in antiviral medicine 2017; 25:55s.

29. Cuomo G, Digaetano M, Menozzi M, et al. Incidence of HCV infection amongst HIV positive men who had sex with men and prevalence data from patients followed at the Infectious Diseases Clinic of Modena, Italy. Digestive and liver disease : official journal of the Italian Society of Gastroenterology and the Italian Association for the Study of the Liver 2018; 50:1334-8.

30. El-Hayek C, Doyle JS, Cuevas M, et al. New hepatitis c infection and re-infection among HIV co-infected men in Melbourne, Australia. Journal of hepatology 2014; 60:S314.

31. Giuliani M, Caprilli F, Gentili G, et al. Incidence and determinants of hepatitis C virus infection among individuals at risk of sexually transmitted diseases attending a human immunodeficiency virus type 1 testing program. Sexually transmitted diseases 1997; 24:533-7.

32. Lachowsky NJ, Stephenson K, Cui Z, et al. Prevalence and factors of HCV infection among HIV-negative and HIV-positive MSM. Topics in antiviral medicine 2016; 24:217.

33. Nishijima T, Shimbo T, Komatsu H, Hamada Y, Gatanaga H, Oka S. Incidence and risk factors for incident Hepatitis C infection among men who have sex with men with HIV-1 infection in a large Urban HIV clinic in Tokyo. Journal of acquired immune deficiency syndromes (1999) 2014; 65:213-7.

34. Pradat P, Huleux T, Raffi F, et al. Incidence of new hepatitis C virus infection is still increasing in French MSM living with HIV. AIDS (London, England) 2018; 32:1077-82.

35. Ruan Y, Jia Y, Zhang X, et al. Incidence of HIV-1, syphilis, hepatitis B, and hepatitis C virus infections and predictors associated with retention in a 12-month follow-up study among men who have sex with men in Beijing, China. Journal of acquired immune deficiency syndromes (1999) 2009; 52:604-10.

36. van der Helm JJ, Prins M, del Amo J, et al. The hepatitis C epidemic among HIV-positive MSM: incidence estimates from 1990 to 2007. AIDS (London, England) 2011; 25:1083-91.

37. van Rooijen M, Heijman T, de Vrieze N, et al. Earlier Detection of Hepatitis C Virus Infection Through Routine Hepatitis C Virus Antibody Screening of Human Immunodeficiency Virus-Positive Men Who Have Sex With Men Attending A Sexually Transmitted Infection Outpatient Clinic: A Longitudinal Study. Sexually transmitted diseases 2016; 43:560-5.

38. Vanhommerig JW, Stolte IG, Lambers FA, et al. Stabilizing incidence of hepatitis C virus infection among men who have sex with men in Amsterdam. Journal of acquired immune deficiency syndromes (1999) 2014; 66:e111-5.

39. Ang L W, Choy C Y, Ng O T, et al. Hepatitis C virus infection in HIV-infected men in Singapore, 2006–2018: incidence and associated factors. Sexual Health, 2021.

40. Garvey L J, Cooke G S, Smith C, et al. Decline in hepatitis C virus (HCV) incidence in men who have sex with men living with human immunodeficiency virus: progress to HCV microelimination in the United Kingdom?. Clinical Infectious Diseases, 2021, 72(2): 233-238.

41. Gonzalez‐Serna A, Macias J, Palacios R, et al. Incidence of recently acquired hepatitis C virus infection among HIV‐infected patients in southern Spain. HIV medicine, 2021, 22(5): 379-386.

42. Gras J, Mahjoub N, Charreau I, et al. Early diagnosis and risk factors of acute hepatitis C in high-risk MSM on preexposure prophylaxis. AIDS, 2020, 34(1): 47-52.

43. Herbert S, Puhr R, Petoumenos K, et al. Characteristics of heterosexually-acquired compared to homosexually-acquired HIV and implications for clinical practice: results from the Australian HIV Observational Database. AIDS care, 2021: 1-7.

44. Ho S Y, Su L H, Sun H Y, et al. Trends of recent hepatitis C virus infection among HIV-positive men who have sex with men in Taiwan, 2011–2018. EClinicalMedicine, 2020, 24: 100441.

45. Hoornenborg E, Coyer L, Boyd A, et al. High incidence of HCV in HIV-negative men who have sex with men using pre-exposure prophylaxis. Journal of hepatology, 2020, 72(5): 855-864.

46. Schmidbauer C, Chromy D, Schmidbauer V, et al. Epidemiological trends in HCV transmission and prevalence in the Viennese HIV+ population. Liver International, 2020, 40(4): 787-796.

47. Tabatabavakili S, Aleyadeh W, Cerrocchi O, et al. Incidence of Hepatitis C Virus Infections Among Users of Human Immunodeficiency Virus Pre-exposure Prophylaxis. Clinical Gastroenterology and Hepatology, 2021.

48. Vuylsteke B, Reyniers T, De Baetselier I, et al. Daily and event‐driven pre‐exposure prophylaxis for men who have sex with men in Belgium: results of a prospective cohort measuring adherence, sexual behaviour and STI incidence. Journal of the International AIDS Society, 2019, 22(10): e25407.
